# Immune monitoring of trabectedin therapy in refractory soft tissue sarcoma patients - the IMMUNYON study

**DOI:** 10.3389/fimmu.2025.1516793

**Published:** 2025-02-11

**Authors:** Paulo Rodrigues-Santos, Jani Sofia Almeida, Luana Madalena Sousa, Patrícia Couceiro, António Martinho, Joana Rodrigues, Ruben Fonseca, Manuel Santos-Rosa, Paulo Freitas-Tavares, José Manuel Casanova

**Affiliations:** ^1^ Laboratory of Immunology and Oncology, Center for Neurosciences and Cell Biology (CNC), University of Coimbra, Coimbra, Portugal; ^2^ Institute of Immunology, University of Coimbra, Faculty of Medicine (FMUC), Coimbra, Portugal; ^3^ Center for Investigation in Environment, Genetics and Oncobiology (CIMAGO), University of Coimbra, Coimbra, Portugal; ^4^ Coimbra Institute for Clinical and Biomedical Research (iCBR), University of Coimbra, Coimbra, Portugal; ^5^ Center for Innovation in Biomedicine and Biotechnology (CIBB), University of Coimbra, Coimbra, Portugal; ^6^ Clinical and Academic Centre of Coimbra (CACC), Coimbra, Portugal; ^7^ Blood and Transplantation Center of Coimbra, Portuguese Institute for Blood and Transplantation (IPST), Coimbra, Portugal; ^8^ Tumor Unit of the Locomotor Apparatus, University Clinic of Orthopedics, Orthopedics Oncology Service, Coimbra Local Health Unit (ULSC), Coimbra, Portugal

**Keywords:** soft tissue sarcoma, trabectedin, immunophenotyping, gene expression profiling, soluble factors, immune checkpoints, progression free survival

## Abstract

Soft tissue sarcomas (STS) encompass over 50 histologic subtypes, representing more than 1% of solid tumors. Standard treatments include surgical resection and therapies such as anthracyclines or trabectedin for advanced cases, though challenges persist due to the tumor microenvironment’s complexity and limited immune profiling data. This study evaluates Trabectedin therapy in 22 refractory STS patients, analyzing progression-free survival (PFS) and immune responses. Immune monitoring included deep immunophenotyping (200+ parameters), gene expression profiling (103 genes), and soluble proteome analysis (99 analytes). Using RECIST1.1 criteria, 68.2% of patients achieved stable disease (SD), while 31.8% exhibited progression disease (PD). Therapy duration revealed 59.1% treated for less than 12 months (<12M) and 40.9% for 12 or more months (≥12M). A significant PFS improvement was observed in SD versus PD patients (p=0.0154), while therapy duration showed no effect (p=0.5433). PD patients showed reduced eosinophils (p<0.05) and Th2 cells (p<0.05). Gene expression analysis identified changes in BTRC (decreased), IFNA1 (increased), and IL9 (increased) in PD versus SD patients (p<0.05). Patients treated ≥12M exhibited increased activated HLA-DR Th2 cells (p<0.05) and decreased exhausted B cells and NK cell subsets (p<0.05). Principal component and hierarchical clustering analyses identified distinct immune profiles associated with RECIST1.1 and therapy duration, underscoring immune profiling’s role in understanding treatment responses. These findings support further research into immune monitoring for future clinical trials.

## 1 Introduction

Soft tissue sarcomas (STS) are a rare and heterogeneous group of diseases. STS comprise over 50 distinct histologic subtypes and represent over 1% of solid tumors^1-3^. This group of diseases affects patients of all ages and can occur anywhere in the body. In addition to their heterogeneity in terms of histology and anatomical localization, STS are highly heterogeneous in terms of molecular characteristics and prognosis (1–3). The standard treatment for localized STS is surgical resection with or without radiotherapy. For locally inoperable or metastatic disease, single-agent anthracycline (4–6) and trabectedin (7, 8) are the first line and second-line treatments, respectively.

Anthracyclines are among the most effective anti-cancer drugs ever developed. Belonging to the anthracycline family, doxorubicin was proved to be active against this group of diseases and changed the therapy for STS patients, whose prognosis was extremely poor (9). Its ability to intercalate the DNA, leading ultimately to cell death, is the primary mechanism responsible for doxorubicin anti-cancer activity. However, the exact mechanism of action is still uncertain (10, 11).

Trabectedin (Yondelis^®^, PharmaMar, Madrid, Spain), initially isolated from the ascidian *Ecteinascidia turbinate*, is a tetrahydroisoquinoline alkaloid now prepared synthetically (12). Like anthracyclines, trabectedin is also a DNA-binding agent. However, the mechanism of action of trabectedin appears to be distinct from the other DNA-damaging drugs available and functions as an immunomodulatory drug (12, 13). Trabectedin exhibits antitumor activity in soft tissue sarcomas by targeting tumor-associated macrophages, selectively depleting pro-tumoral macrophages while sparing anti-tumoral ones, thus altering the tumor microenvironment (14).

Despite the remarkable improvement in STS diagnosis and treatment, their rarity and diversity result in a challenging diagnosis as well as a limited response to therapy (15, 16). Moreover, the lack of effectiveness of current therapies can also be attributed to the complexity of the tumor microenvironment (TME) that comprises different populations of non-tumor cells, including immune cells.

With the development of immunotherapy, the assessment of the immunological status of cancer patients is growing in importance since the immune cells and the mediators of the immune response may represent potential immunotherapeutic agents or targets, or potential biomarkers for an accurate prognosis and to monitor the response to therapy.

Although initially STS were simply classified as ‘non-immunogenic’ tumors, studies have proved that this characterization does not apply to all (17). Indeed, different TME compositions have been found in STS patients, and some of them exhibited an elevated infiltration of immune cells and immune-related factors. Furthermore, the TME has also been associated with patient prognosis and could predict the patient’s response to therapy (17, 18).

Despite these advances, the rarity and heterogeneity of STS make TME evaluation challenging, often requiring invasive biopsies that limit the availability of samples and longitudinal studies. Peripheral immune status assessment through blood biomarkers offers a minimally invasive alternative, enabling dynamic immune monitoring. Systemic inflammatory indices, such as the lymphocyte-to-monocyte ratio (LMR) and neutrophil-to-lymphocyte ratio (NLR), have emerged as significant prognostic markers in STS (19). Elevated LMR has been associated with improved clinical outcomes, serving as a reliable predictor of treatment response and overall survival in STS patients (20). In contrast, a low LMR correlates with worse prognosis, reflecting an imbalance in immune homeostasis and a pro-tumor inflammatory environment. LMR has been shown to improve predictive accuracy and clinical outcomes in STS patients, highlighting its potential for aiding treatment stratification. Similarly, NLR has been found to be significantly associated with survival in synovial sarcoma patients, underscoring its prognostic importance (21). Furthermore, systemic inflammatory indices in STS patients treated with trabectedin identified LMR as a crucial marker linked to improved treatment responses (22). These findings emphasize the clinical value of inflammatory indices as minimally invasive biomarkers to predict treatment efficacy and patient outcomes in STS management.

Further research has identified correlations between immune cell subsets, such as T cells and natural killer (NK) cells, with patient outcomes. For example, increased myeloid-derived suppressor cells (MDSCs) in the blood of STS patients have been linked to reduced NK cell function and poor prognosis (23–25). Similarly, alterations in immune-related genes and soluble factors have been shown to reflect immune dysregulation in STS (26, 27). Recently, our group demonstrated the benefits of using deep imunophenotyping, transcriptomic analysis, and soluble proteomics to identify peripheral immune biomarkers and signatures associated with survival in soft tissue sarcoma (28).

Despite these promising findings, the results are often inconsistent due to variations in patient cohorts, therapies, and methodologies (29). Therefore, further studies integrating multi-parametric immune profiling are essential to better understand the immunological landscape of STS and develop immune-based prognostic tools and therapies.

The aim of this study was to assess the peripheral immunological status, including the immune cells, immune-related genes, immune-related soluble factors, and immune checkpoints of STS patients, characterizing the STS patients with a better response to trabectedin therapy. Immune monitoring revealed key markers, including decreased eosinophils, Th2 cells, and dendritic cells in patients with disease progression, along with significant gene expression changes (BTRC, IFNA1, IL9). Additionally, prolonged therapy (>12 months) was linked to alterations in specific immune subsets, such as activated NK cells and HLA-DR-positive T cells, highlighting potential biomarkers of response.

## 2 Material and methods

### 2.1 Study design

This was a non-interventional, prospective study where trabectedin was administered according to the summary of product characteristics (SmPC) in the local language. There was no involvement with any treatment decisions for the patients included in the study. The choice of therapy was made prior to the patient’s inclusion in the study. Treatment administration was independent of and dissociated from participation in the study.

### 2.2 Selection criteria

Patients meeting the inclusion criteria were invited to participate in the study. Before any study-specific data was recorded, patients were provided with all the study details prior to their decision to participate and were requested to sign an informed consent form (ICF) prior to their participation in the study.

#### 2.2.1 Inclusion criteria

Patients had to comply with all the criteria to be enrolled in the study. They had to be 18 years or older and have histologically proven STS. The patients needed to have advanced STS and measurable disease. A baseline tumor evaluation for RECIST 1.1 (30) and/or Choi criteria must have been performed, with assessments completed by available methods. The baseline imaging procedure should have been the one conducted within 30 days prior to the initial trabectedin administration (first treatment cycle) or the earliest imaging following the start of trabectedin therapy, and this baseline image had to be available. Patients must have completed at least one cycle of trabectedin according to the SmPC before being included in the study. They were required to have been currently receiving trabectedin as per the SmPC, treated according to the indication in line with the local label and reimbursement for trabectedin treatment, and demonstrate evidence of adequate end-organ function as outlined in the SmPC. Lastly, patients must have signed an ICF, acknowledging that they understood the purpose and procedures of the study and were willing to participate.

#### 2.2.2 Exclusion criteria

Patients who met any of the following criteria were excluded from participating in the study: those with contraindications for the use of trabectedin as defined in the SmPC, those currently receiving any other therapy for STS, those who had not recovered from prior treatment-related toxicity (except for alopecia), and those undergoing a rechallenge of trabectedin.

### 2.3 Study samples

Peripheral blood samples and clinical data were collected at the Tumor Unit of the Locomotor Apparatus (UTAL) - Orthopedic Service from Coimbra Hospital and Universitary Centre (CHUC), Portugal, from June 2019 to December 2022.

This work follows the World Medical Association’s Helsinki Declaration for human-subject research. All the volunteers agreed and signed informed consent to participate. The present study was approved by the Service Directors, the Ethical Committee (CHUC-021-19 from 30 May 2019), and the Administration Board of the Coimbra Hospital and Universitary Centre (CHUC), Coimbra, Portugal.

The study group consisted of 22 patients with STS treated with Trabectedin (Yondelis^®^, PharmaMar, Madrid, Spain), and the demographic and clinical-pathological characterization at baseline are presented in **Table 1**. Samples were collected 42 ± 34 months after diagnostic and STS patients were followed for an average of 15 ± 2 months.

**Table 1:** Baseline characteristics of Soft Tissue Sarcoma (STS) patients enrolled in the study.

Characteristic	STS Trabectedin (N=22)
Median Age (range), years	55 (29-78)
Sex, n, (%)	
* Female*	*12 (54.5)*
* Male*	*10 (45.5)*
Histology Classification, n, (%)	
* 8890/3 Leiomyosarcoma, NOS*,	*9 (40.9)*
* 9040/3 Synovial sarcoma. NOS*	*3 (13.6)*
* 8858/3 Dedifferentiated liposarcoma*	*2 (9.1)*
* 8805/3 Undifferentiated sarcoma*	*2 (9.1)*
* 8811/3 Fibromyxosarcoma*	*1 (4.5)*
* 8850/3 Liposarcoma. NOS*	*1 (4.5)*
* 8854/3 Pleomorphic liposarcoma*	*1 (4.5)*
* 9041/3 Synovial sarcoma. spindle cell*	*1 (4.5)*
* 9044/3 Clear cell sarcoma. NOS*	*1 (4.5)*
* 9540/3 Malignant peripheral nerve sheath tumor*	*1 (4.5)*
Anatomic Location, n, (%)	
* Axial*	
* C17.1 Jejunum*	*1 (4.5)*
* C38 Malignant neoplasm of heart, mediastinum and pleura*	*1 (4.5)*
* C47.3 Peripheral nerves of thorax*	*1 (4.5)*
* C48.0 Retroperitoneum*	*5 (22.7)*
* C49.3 Connective and soft tissue of thorax*	*2 (9.1)*
* C49.4 Connective and soft tissue of abdomen (Hypochondrium)*	*1 (4.5)*
* C49.5 Connective and soft tissue of pelvis*	*1 (4.5)*
* C49.6 Connective and soft tissue of trunk, unspecified*	*1 (4.5)*
* Limbs*	
* C49.2 Connective and soft tissue of lower limb, including hip*	*6 (27.3)*
* Gynecologic*	
* C55.9 Utero, SOE*	*2 (9.1)*
Genitourinary	
* C63.1 Spermatic cord*	*1 (4.5)*
Disease Stage, n, (%)	
Primary tumor	4 (18.2)
Local recurrence	1 (4.5)
Metastatic disease	15 (68.2)
Local recurrence and metastatic disease	2 (9,1)
Therapy, n, (%)	
Anthracycline-based therapy 1^st^ line, Trabectedin 2^nd^ line	12 (54.4)
Trabectedin-based therapy 1^st^ line	8 (36.4)
Trabectedin + others	2 (9.1)

**Figure 1:**
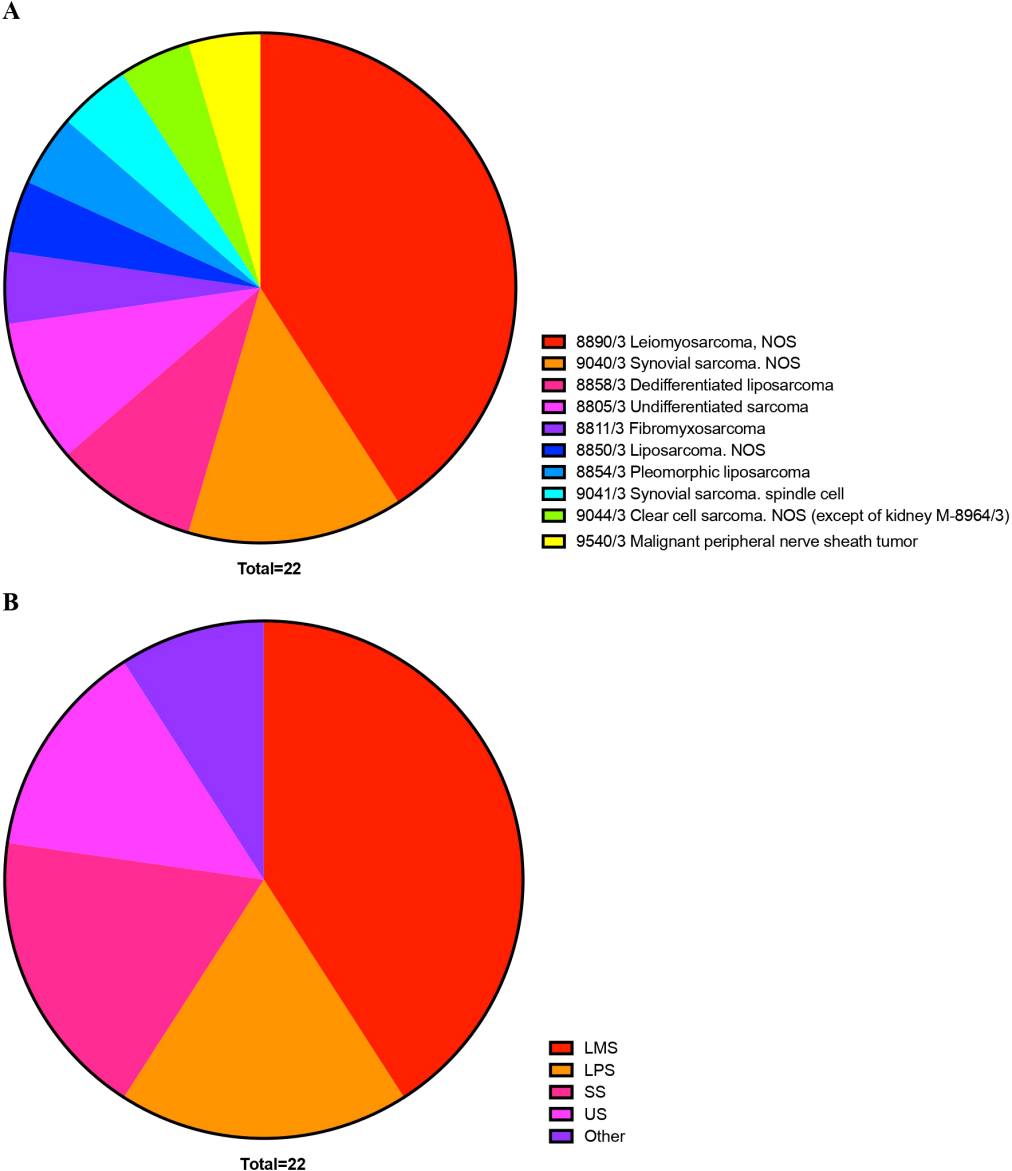
Distribution of Soft Tissue Sarcoma patients participating in the study, according to histology **(A)** and histology group **(B)**, following to the International Classification of Diseases for Oncology, Third Edition (ICD-O-3).

Histologic classification at baseline according to the International Classification of Diseases for Oncology, Third Edition (ICD-O-3) is presented in **Figures 1A, B**.

### 2.4 Immunophenotyping

Blood samples were drawn at the baseline, after cycle 1 and subsequent visits, and during the follow-up period. Peripheral blood samples collected into EDTA were counted with a hematological counter (DxH500, Beckman Coulter, Pasadena, CA, USA). Whole blood, 100 µL of or up to 1 x 10^6^ cells, were incubated with a panel of monoclonal antibodies (**Supplementary Files S1, S2**) (31, 32) for 15 minutes in the dark at room temperature. Then, red blood cells were lysed with BD Lysing Solution 1x (BD Biosciences, San Jose, CA, USA) for 10 minutes in the dark. The cell suspensions were centrifuged at 450 x g for 5 minutes, and the supernatants were discarded. The suspensions were washed with phosphate-buffered saline (PBS), and lastly, samples were acquired in a BD FACSCanto II eight-color flow cytometer (BD Biosciences, San Jose, CA, USA) using BD FACSDiva 6.0 software (Becton Dickinson, San Jose, CA, USA). Gating strategy for the analysis of T, B, NK cells, monocytes, dendritic cells, and myeloid-derived suppressor cells is presented as a supplement (**Supplementary File S3**). All the data were treated with FlowJo^®^ v.10.7 (BD Life Sciences, Ashland, OR, USA).

### 2.5 Immune-related gene expression (real-time quantitative PCR)

Whole blood samples were collected into PAXgene^®^ Blood RNA tubes (PreAnalytiX GmbH, Hombrechtikon, Switzerland), and RNA extracted using the PAXgene Blood RNA Kit (PreAnalytiX GmbH, Hombrechtikon, Switzerland) according to the manufacturer’s instructions. Purity of the RNA was assessed using a NanoDrop 2000 (ThermoFisher Scientific, Wilmington, DE, USA). Complementary DNA was synthesized with the iScriptTM Reverse Transcription Supermix for RT-qPCR (BIO-RAD, Hercules, CA, USA).

The real-time quantitative PCR was performed using iTaqTM Universal SYBR^®^ Green Supermix (BIO-RAD, Hercules, CA, USA), according to the manufacturer´s instructions. In short, the optimized ready-to-use reaction master mix was combined with the forward and reverse primers for the gene of interest or reference genes (33). All oligonucleotide primer sequences are presented in **Supplementary File S4**. Then, equal aliquots were dispensed into the plate containing the cDNA samples and non-template controls to guarantee no unspecific amplifications. Lastly, the plates were incubated in a thermal cycler Roche LightCycler II 480 (Roche, Basel, Switzerland) previously programmed for: one pre-incubation cycle of 2 minutes at 95°C, 50 amplification cycles of 5 seconds at 95°C and one minute at 60°C, and a melt curve analysis (65-95°C). The results were analyzed with qBase+ v3.2 software (Biogazelle, Gent, Belgium).

### 2.6 Multiplex Analyte Profiling

The plasma was isolated from the whole blood, collected into EDTA tubes, centrifuged at 1250 x g for 10 minutes, and then stored at −20°C until analysis. Plasmatic levels of several cytokines, chemokines, growth factors, and immune checkpoints were measured using: ProcartaPlex Human Immune Monitoring 65-Plex Panel (Cat. Nr. EPX650-10065-901, Invitrogen), ProcartaPlex Human Immuno-Oncology Checkpoint Panel 1 14-Plex (Cat. Nr. EPX14A-15803-901, Invitrogen), ProcartaPlex Human Immuno-Oncology Checkpoint Panel 2 14-Plex (Cat. Nr. EPX140-15815-901, Invitrogen), and ProcartaPlex Human Immuno-Oncology Checkpoint Panel 3 10-Plex (Cat. Nr. EPX100-15820-901, Invitrogen), all from ThermoFisher Scientific, Waltham, Massachusetts, USA (**Supplementary Files S5, S6**). All the multiplex immunoassays were performed according to the manufacturer’s instructions and acquired on the Luminex^®^ 100/200™ xMAP™ System (Luminex Corporation, Austin, Texas, USA). The run data were analyzed using the ProcartaPlex™ Analysis App (ThermoFisher Scientific, Waltham, Massachusetts, USA). The analytes with concentrations outside the limits of quantification were excluded from the analysis.

### 2.7 Statistical analysis

All statistical analyses and the graphs were performed and generated using GraphPad Prism 10.4.1 for Windows (GraphPad Software, San Diego, CA, USA). Multiple Mann-Whitney tests were used to compare the means between two groups. The data is presented as mean ± standard deviation, and a value of p < 0.05 was considered statistically significant. For the visualization of clusters of multivariate data, we used the Principal Component Analysis (PCA) and heatmaps, accessed online from ClustVis (34) (https://biit.cs.ut.ee/clustvis). In the PCA analysis, the original values were ln(x+1)-transformed, unit variance scaling was applied to rows, and singular value decomposition (SVD) with imputation was used to calculate the principal components. The X and Y axes show principal component 1 and principal component 2 that explain the percentages indicated of the total variance. Prediction ellipses are such that with a probability of 0.95, a new observation from the same group will fall inside the ellipse. For the heatmaps, the original values are also ln(x+1)-transformed, rows are centered, unit variance scaling is applied to rows, imputation is used to estimate missing values, and both rows and columns are clustered using Manhattan distance and Ward (unsquared distances) linkage. Clustering distances were obtained using Pearson correlation subtracted from 1. The Ward linkage method was calculated using the sum of squared differences from points to centroids as the distance. For the OS analysis, we used the Kaplan-Meier survival analysis. OS time was defined as the time from the sample harvest to the date of death or the date of the last follow-up (censored patients). The Kaplan–Meier curves were performed in IBM SPSS statistics version 26.0 (IBM Corp., Armonk, NY, USA).

### 2.8 Bioinformatic analyses

Clinical, genomic, and transcriptomic data from 206 primary sarcoma samples in The Cancer Genome Atlas (TCGA) were analyzed using CBioPortal v6.0.22 (35). The dataset ‘Adult Soft Tissue Sarcomas’ (36) was selected, and 174 soft tissue sarcoma samples were included in the survival analysis, excluding those without complete genomic and expression data, as well as uterine sarcoma and nerve sheath tumor samples. CBioPortal was additionally utilized to visualize data from the TCGA sarcoma cohort, retrieve patient clinical information, and access genomic and transcriptomic data.

## 3 Results

### 3.1 Progression-free survival and duration of trabectedin therapy

**Figure 2:**
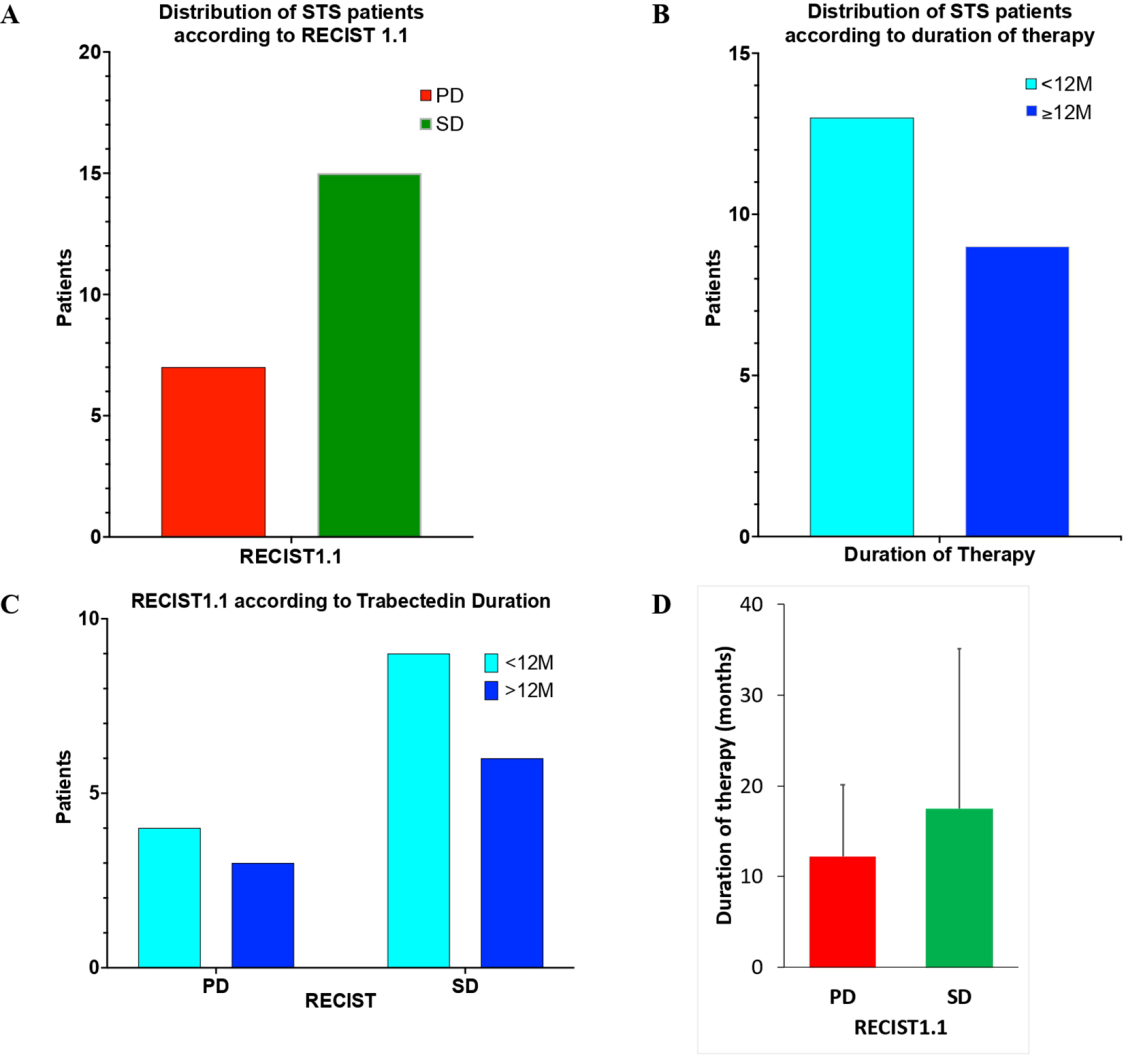
Distribution of Soft Tissue Sarcoma patients of the study according to RECIST1.1 **(A)**; duration of trabectedin therapy **(B)**; RECIST1.1 according to duration of Trabectedin therapy **(C)**; and duration of trabectedin therapy (in months) according to RECIST1.1 **(D)**.

The primary goal of this study was to analyze progression-free survival (PFS) in STS patients treated with trabectedin using RECIST1.1). Distribution of the STS participants is shown in **Figure 2A**. Stable disease (SD) was achieved for 68.2% (15/22) of STS patients treated with Trabectedin. Progression disease (PD) was observed in 31.8% (7/22) of the patients.

In addition, the duration of Trabectedin was also analyzed (**Figure 2B**). Patients with less than 12 months (<12M) of Trabectedin therapy represented 59.1% (13/22) and those with 12 or more months (≥12M) were 40.9% (9/22).

The analysis of the combined distribution of RECIST1.1 and duration of Trabectedin therapy is presented in **Figures 2C, D**. No statistically significant difference (two-sided Mann-Whitney test, p=0.6667) was observed for RECIST1.1 according to duration of Trabectedin therapy.

**Figure 3:**
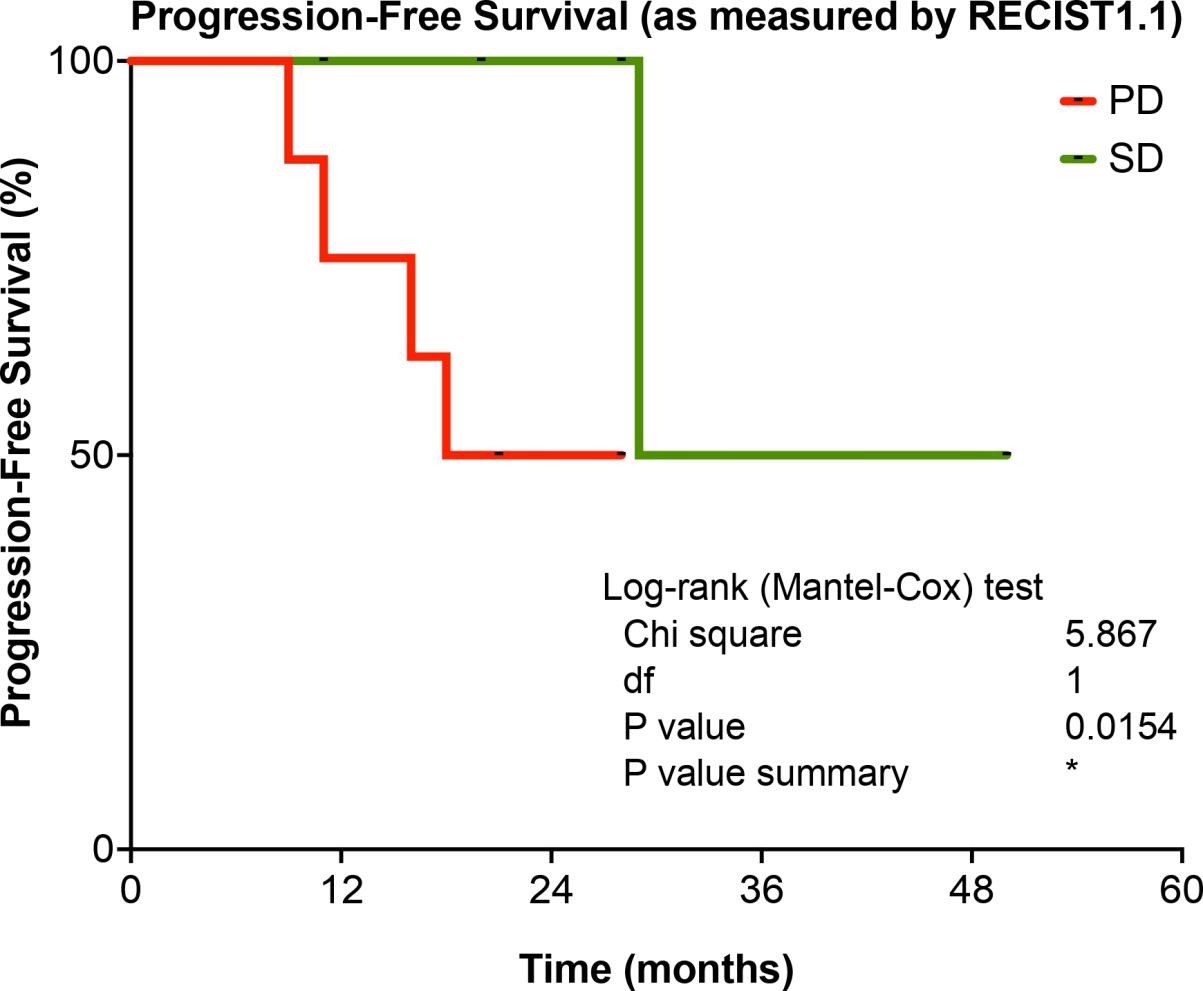
Progression-Free Survival (PFS) according to therapy response according to RECIST1.1, for STS patients in the study. PD, Progression Disease; SD, Stable Disease. *p-value < 0.05.

Progression-free survival (PFS), as measured by RECIST1.1 is present in **Figure 3**. A significant increase (p=0.0154) is observed for STS patients that achieve stable disease (SD) under Trabectedin therapy when compared with those that were classified with progression disease (PD).

**Figure 4:**
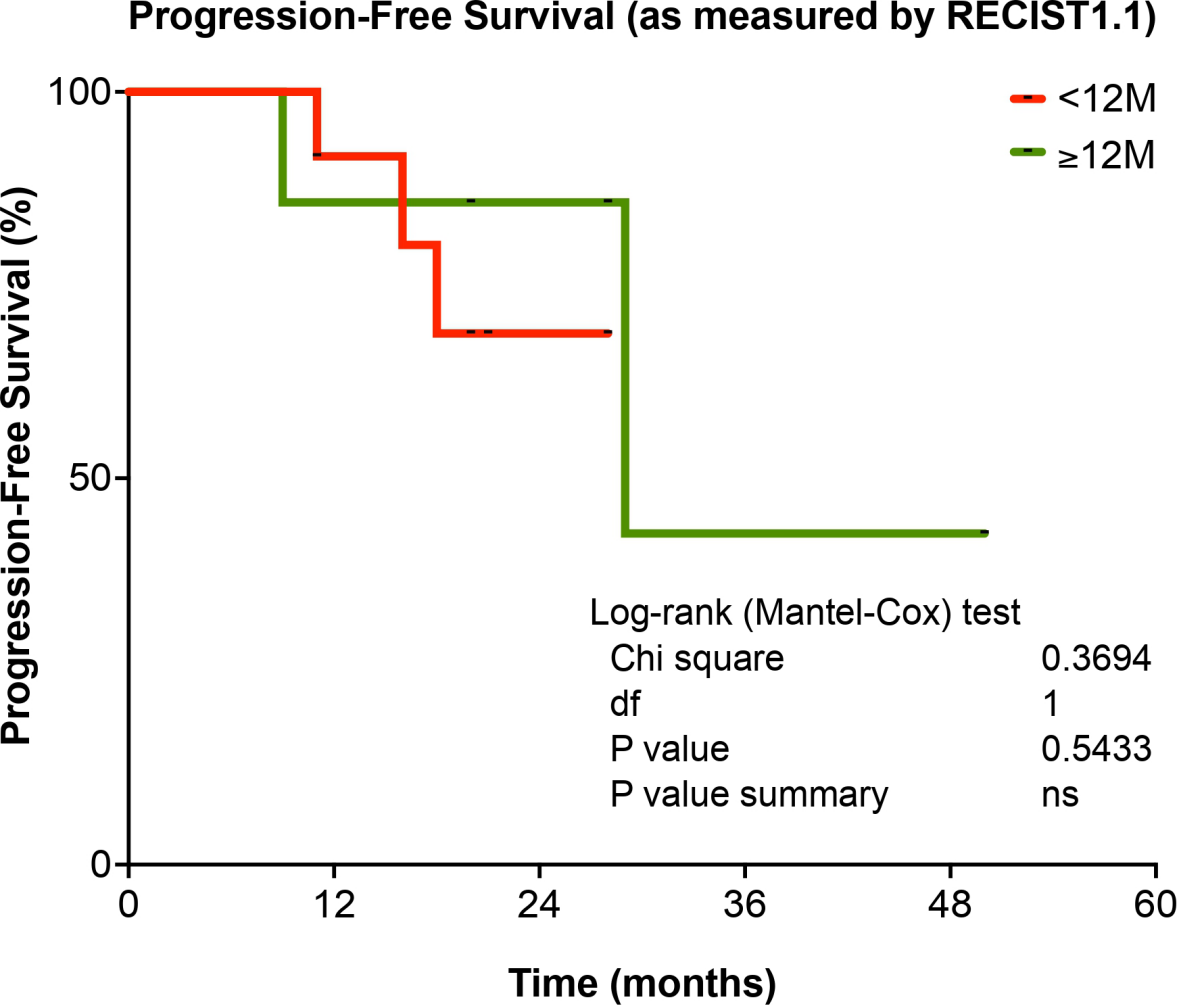
Progression-Free Survival (PFS), according to duration of Trabectedin therapy, for STS patients in the study. <12M, Trabectedin therapy for less than 12 months; ≥12M, Trabectedin therapy for 12 or more months. ns, non-significant.

PFS according to duration of Trabectedin therapy is present in **Figure 4**. No significant difference (p=0.5433) was observed for STS patients according to duration of Trabectedin therapy, when comparing those with less than 12 months (<12M) with STS patients that underwent treatment for 12 months or more (≥12M).

### 3.2 Immune monitoring of trabectedin therapy in refractory soft tissue sarcoma patients

In the present study, one of the aims was the immune monitoring of Trabectedin therapy in refractory STS patients to establish additional efficacy parameters of different therapeutic strategies.

Using complementary approaches to characterize the peripheral immune response of STS patients to Trabectedin therapy, deep immunophenotyping (more than 200 immune cell parameters), immune-related gene expression profiling (103 genes), and soluble proteome analysis (99 analytes including cytokines, chemokines, growth factors, and soluble immune checkpoints) were performed for this study.

#### 3.2.1 Immune monitoring of trabectedin therapy and progression-free survival

**Figure 5:**
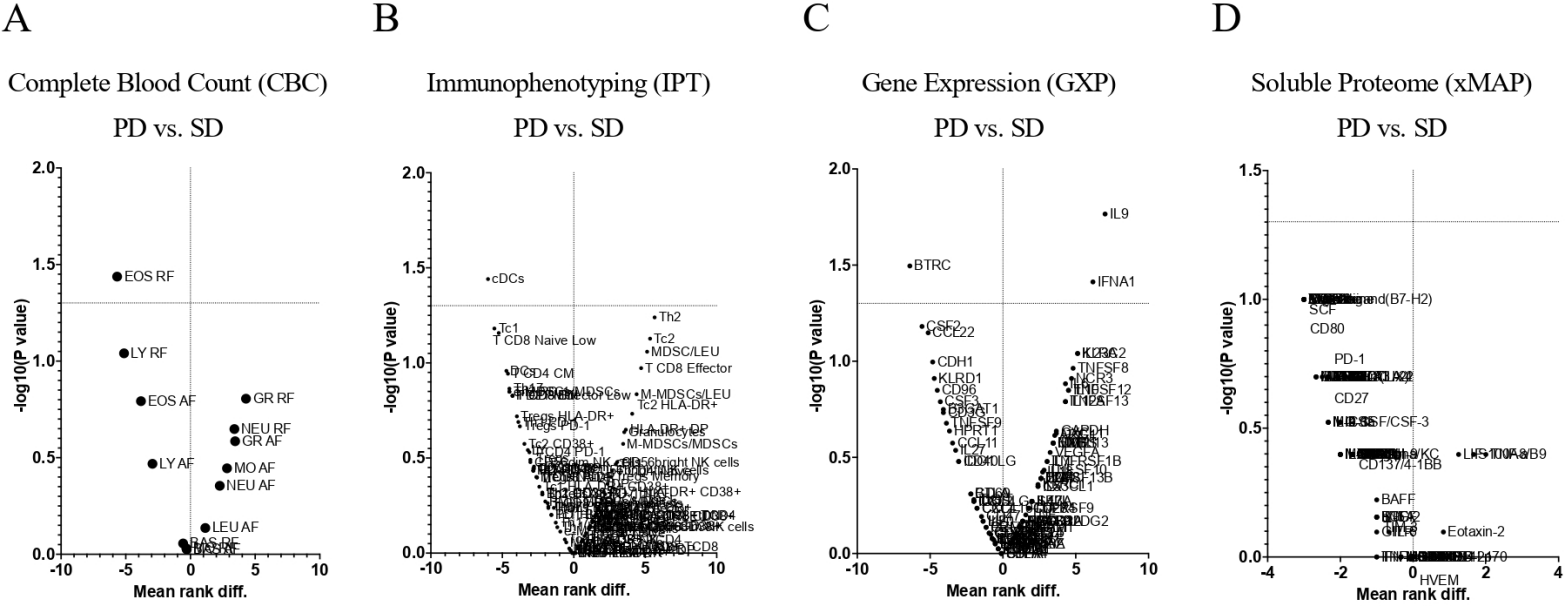
Volcano plots of Soft Tissue Sarcoma patients according to RECIST1.1. Comparisons of PD vs. SD for Complete Blood Count **(A)**, Immunophenotyping **(B)** Immune-Related Gene Expression **(C)** and Soluble Proteome **(D)**. CBC, Complete Blood Count; IPT, Immunophenotyping; GXP, Gene Expression; xMAP, x Multi-Analyte Profiling; PD, Progression Disease; SD, Stable Disease.

A scatterplot (volcano plot) was used to quickly identify changes in the large data set composed of replicate data obtained with this project. In **Figure 5**, a volcano plot of STS patients, according to RECIST1.1, shows the significance vs. fold change (mean rank difference) on the y and x axes, respectively. Comparisons of PD vs. SD are presented for complete blood count (CBC), immunophenotyping (IPT), immune-related gene expression (GXP), and soluble proteome (xMAP).

Complete Blood Count revealed that the relative frequency of eosinophils was found significantly decrease (p <0.05) in PD patients when compared to SD patients (**Figure 5A**).

**Figure 6:**
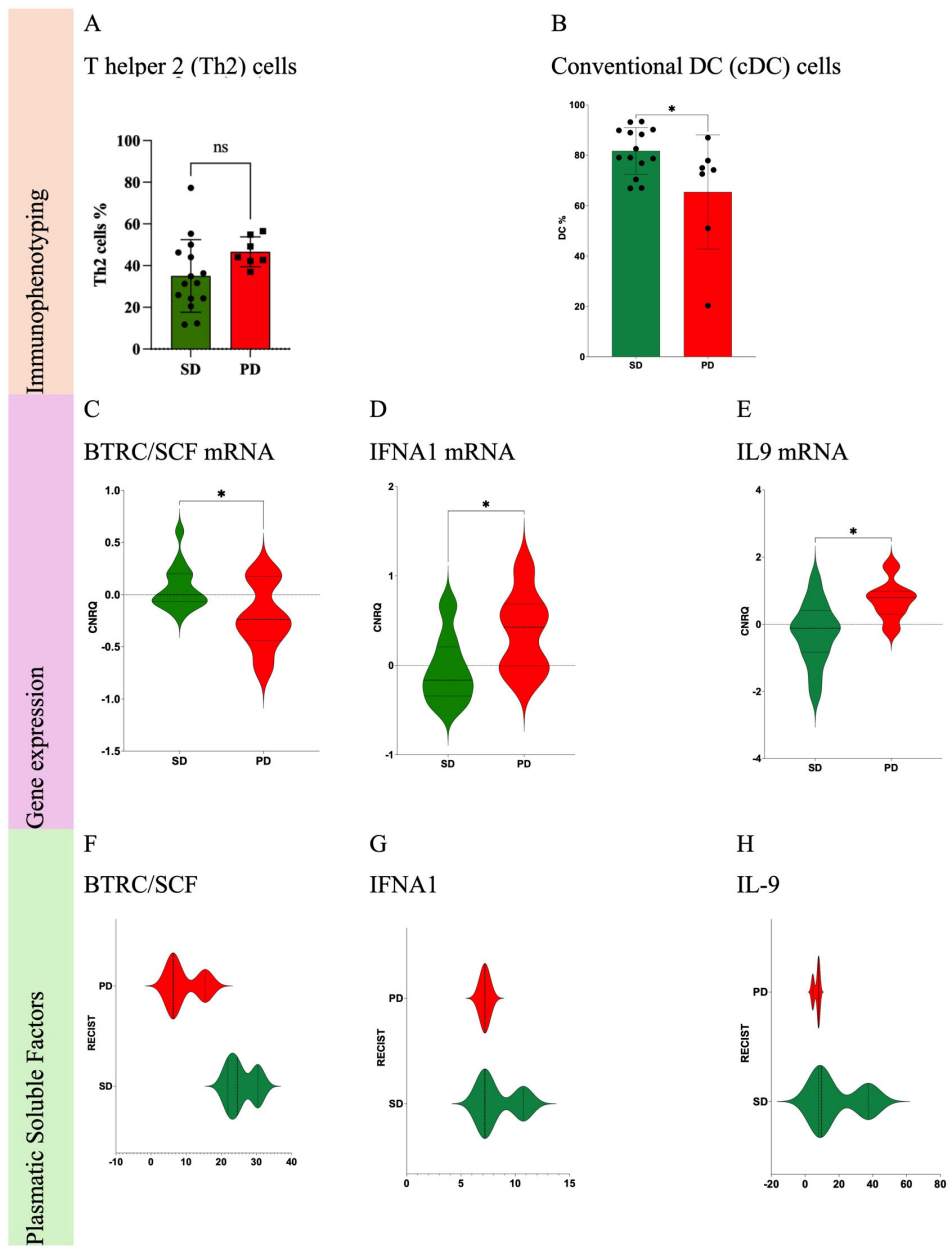
Statistically significant differences of immune cells from Soft Tissue Sarcoma patients according to RECIST1.1. Comparisons of PD vs. SD for immune cells: Th2 cells **(A)** and cDC **(B)**. Comparisons of PD vs. SD for immune-related gene expression: BTRC/SCF mRNA **(C)**, IFNA1 mRNA **(D)** and IL9 mRNA **(E)**. Comparisons of PD vs. SD for plasmatic soluble factors: BTRC/SCF **(F)**, IFNA1 **(G)** and IL9 **(H)**. PD, Progression Disease; SD, Stable Disease; *p-value < 0.05. ns, non-significant.

Immunophenotyping (**Figure 5B**) showed an increase (p=0.0576) for type 2 helper T cells (Th2) (**Figure 6A**) and a decrease (p=0.0362) for cDCs (**Figure 6B**).

Immune-related gene expression profiling (**Figure 5C**) allowed the identification of 3 genes with significant differences (p <0.05) when comparing PD vs. SD patients: decrease of beta-transducin repeat containing (BTRC) gene, **Figure 6C**; increase of interferon alpha-1 (IFNA1), **Figure 6D**; and interleukin 9 (IL9) genes, **Figure 6E**.

Soluble proteome analysis (**Figure 5D**) disclosed any association (**Figures 6F-H**) of the 99 targets with PFS, as measured by RECIST1.1.

#### 3.2.2 Immune monitoring and duration of trabectedin therapy

**Figure 7:**
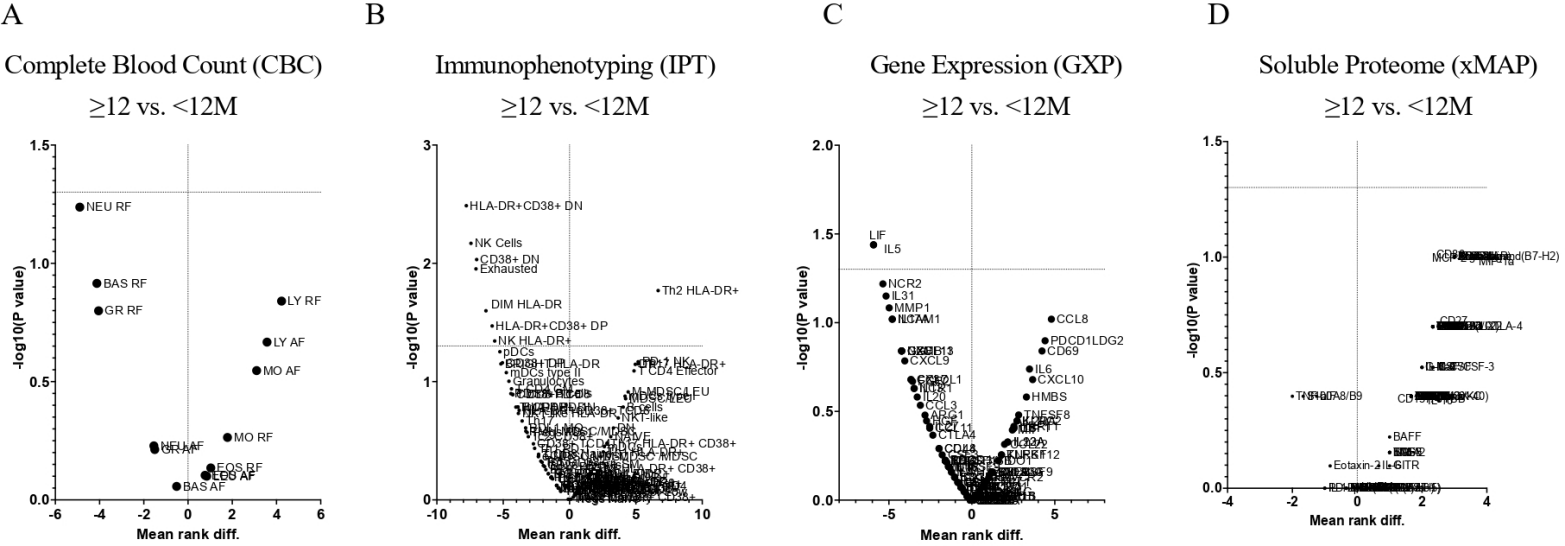
Volcano plots of Soft Tissue Sarcoma patients according to Trabectedin therapy duration in months. Comparisons of <12M vs. ≥12M for Complete Blood Count **(A)**, Immunophenotyping **(B)** Immune-Related Gene Expression **(C)** and Soluble Proteome **(D)**. CBC, Complete Blood Count; IPT, Immunophenotyping; GXP, Gene Expression; xMAP, x Multi-Analyte Profiling; <12M, Trabectedin therapy for less than 12 months; ≥12M, Trabectedin therapy for 12 or more months.

In **Figure 7**, a volcano plot compares STS patients treated with Trabectedin therapy for less than 12 months (<12M) with those with 12 months or more (≥12M) for complete blood count (CBC), immunophenotyping (IPT), immune-related gene expression (GXP), and soluble proteome (xMAP).

Complete Blood Count revealed (**Figure 7A**) no significant decrease (p > 0.05) in STS patients treated with Trabectedin for less than 12 months (<12M) when compared to those with 12 or more months (≥12M).

**Figure 8:**
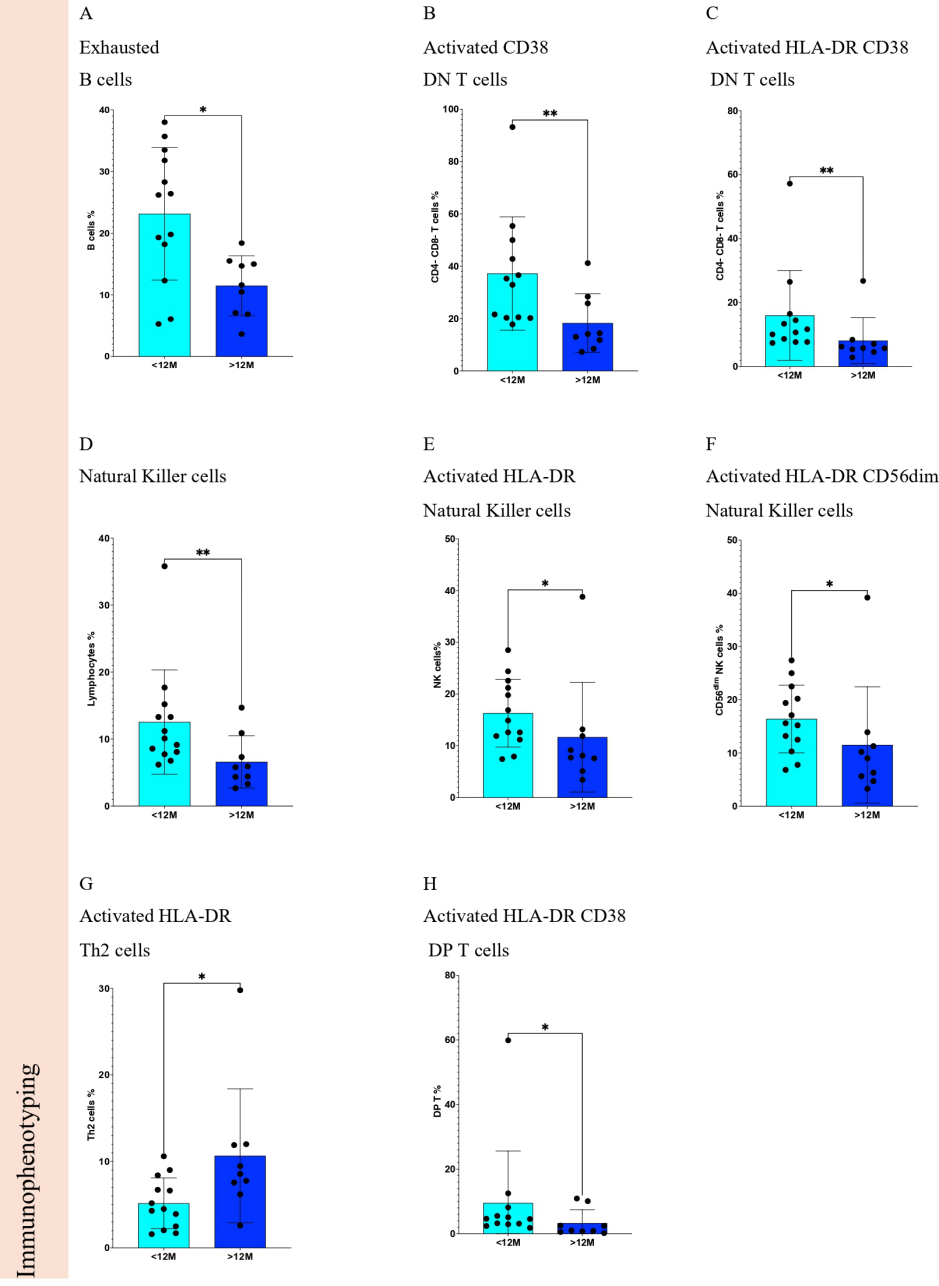
Statistically significant differences of immune cells from Soft Tissue Sarcoma patients according to Trabectedin therapy duration in months. Comparisons of <12M vs. ≥12M for Immunophenotyping for **(A)** exhausted B cells, **(B)** activated CD38 DN T cells, **(C)** activated HLA-DR CD38 DN T cells, **(D)** Natural killer cells, **(E)** activated HLA-DR Natural killer cells, **(F)** activated HLA-DR CD56dim Natural killer cells, **(G)** activated HLA-DR Th2 cells, and **(H)** activated HLA-DR CD38 DP T cells. <12M, Trabectedin therapy for less than 12 months; ≥12M, Trabectedin therapy for 12 or more months. *p-value < 0.05; **p-value < 0.01.

Immunophenotyping (**Figure 7B**) showed a significant decrease (p <0.05) for exhausted B cells, CD19+ CD20+ IgD- CD27-, (**Figure 8A**), activated CD38 double-negative (DN) T cells, CD3+ CD4- CD8- CD38+, (**Figure 8B**), activated HLA-DR CD38 DN T cells, CD3+ CD4- CD8- HLA-DR+ CD38+, (**Figure 8C**), Natural Killer (NK) cells, CD3- CD56+, (**Figure 8D**), activated HLA-DR NK cells, HLA-DR+ CD3- CD56+, (**Figure 8E**), activated HLA-DR CD56dim NK cells, HLA-DR+ CD3- CD56 dim, (**Figure 8F**), and activated HLA-DR CD38 double-positive (DP) T cells, CD3+ CD4+ CD8+ HLA-DR+ CD38+, (**Figure 8H**), and a significant increase of activated HLA-DR Th2 cells, HLA-DR+ CD3+ CXCR3- CCR6-, (**Figure 8G**) when comparing STS patients treated ≥12M with Trabectedin with those with <12M.

**Figure 9:**
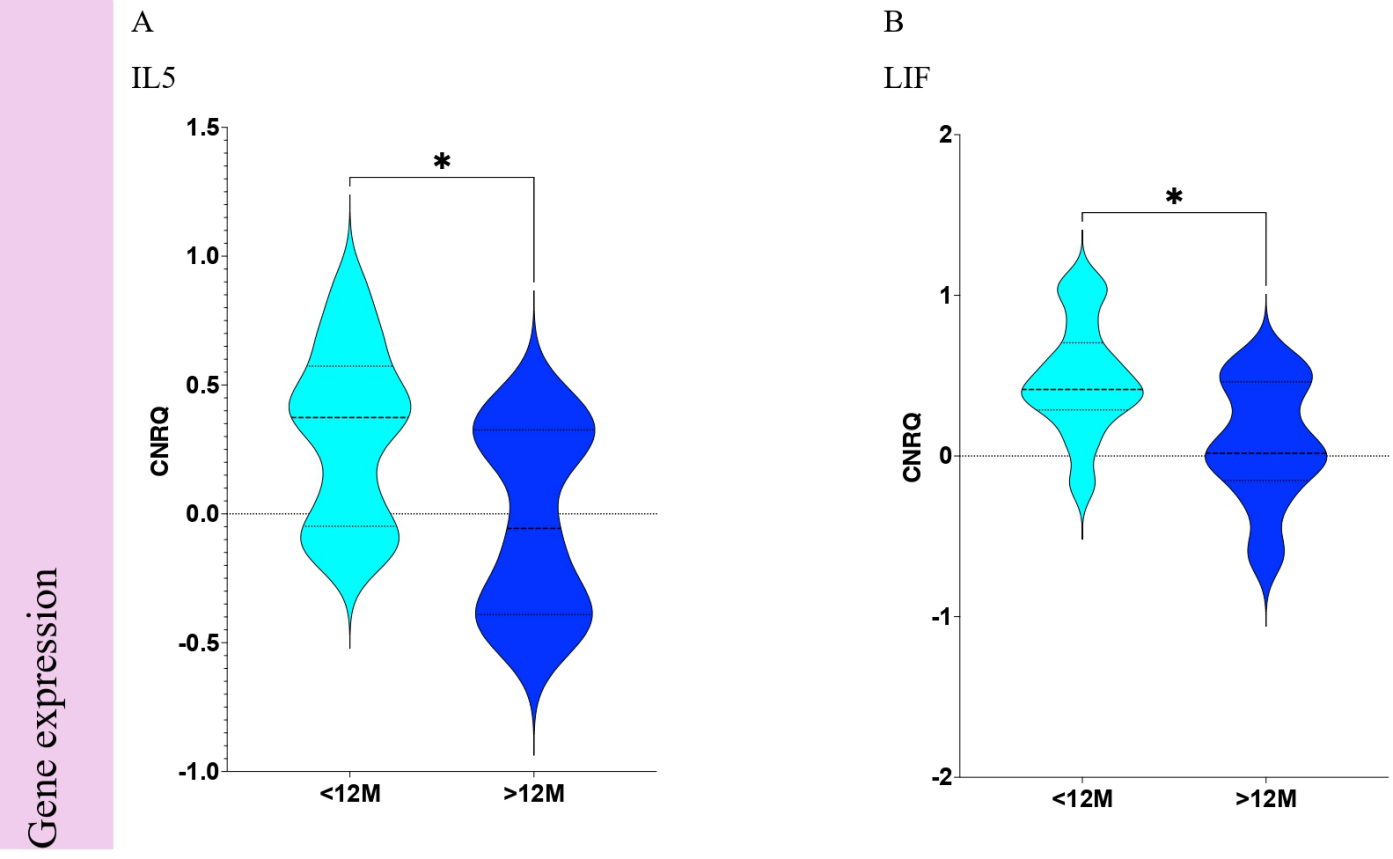
Statistically significant differences of immune-related gene expression from Soft Tissue Sarcoma patients according to Trabectedin therapy duration in months. Comparisons of <12M vs. ≥12M for immune-related gene expression of **(A)** IL5 and **(B)** LIF. <12M, Trabectedin therapy for less than 12 months; ≥12M, Trabectedin therapy for 12 or more months. *p-value < 0.05.

Immune-related gene expression profiling (**Figure 7C**) allowed the identification of 2 genes with significant differences (p <0.05) when comparing <12M vs. ≥12M STS patients: decrease of interleukin 5 (IL5) gene (**Figure 9A**) and leukemia inhibitory factor (LIF) gene (**Figure 9B**).

**Figure 10:**
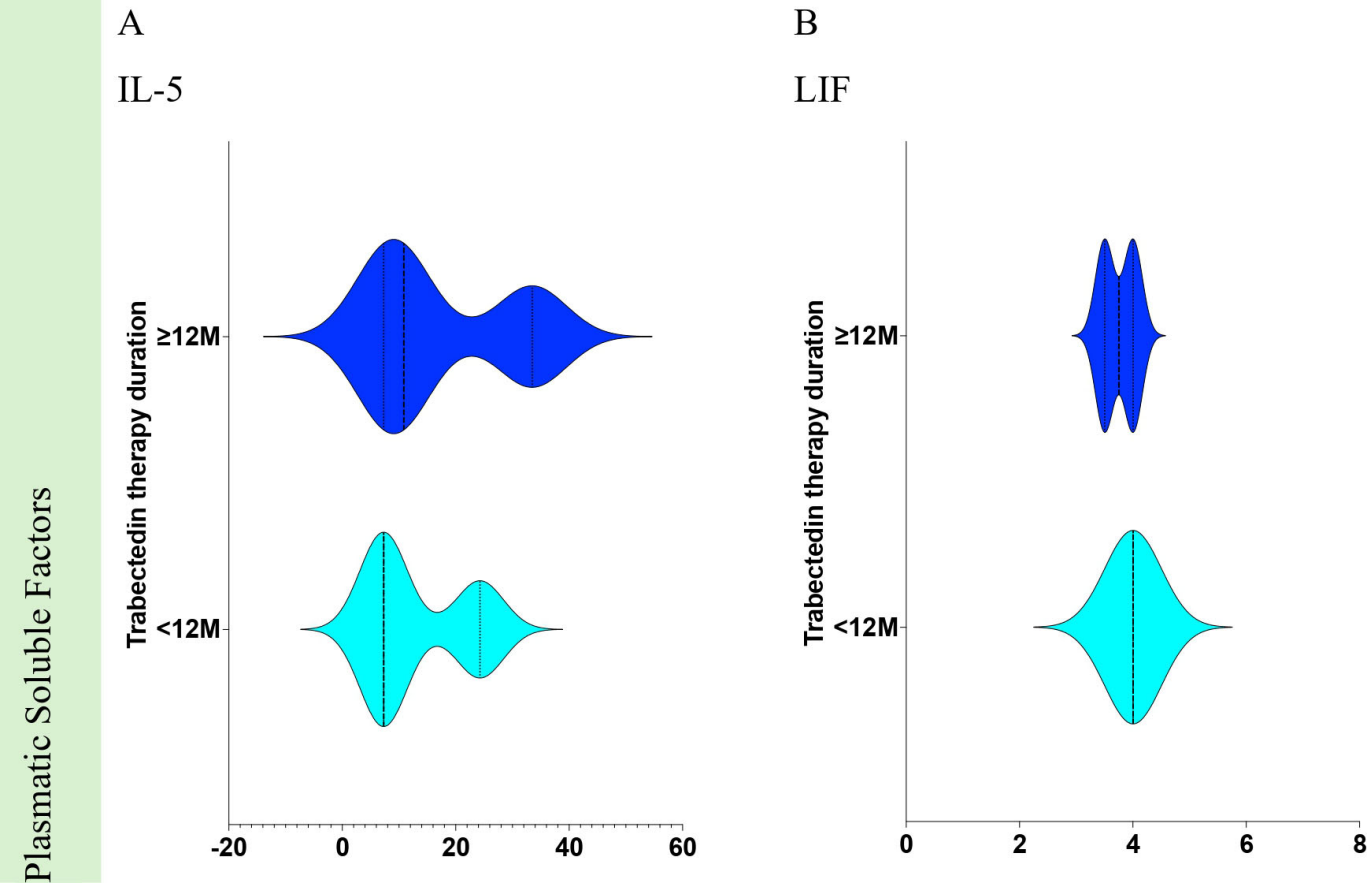
Statistically significant differences of plasmatic soluble factors from Soft Tissue Sarcoma patients according to Trabectedin therapy duration in months. Comparisons of <12M vs. ≥12M for plasmatic soluble factors for **(A)** IL-5 and **(B)** LIF. <12M, Trabectedin therapy for less than 12 months; ≥12M, Trabectedin therapy for 12 or more months.

Soluble proteome analysis disclosed any association (**Figures 7D**) of the 99 targets when comparing <12M vs. ≥12M STS patients. The equivalent proteins for genes that revealed associations are presented for IL-5 (**Figure 10A**) and LIF (**Figure 10B**).

### 3.3 Combined analysis of progression-free survival and trabectedin therapy duration

**Figure 11:**
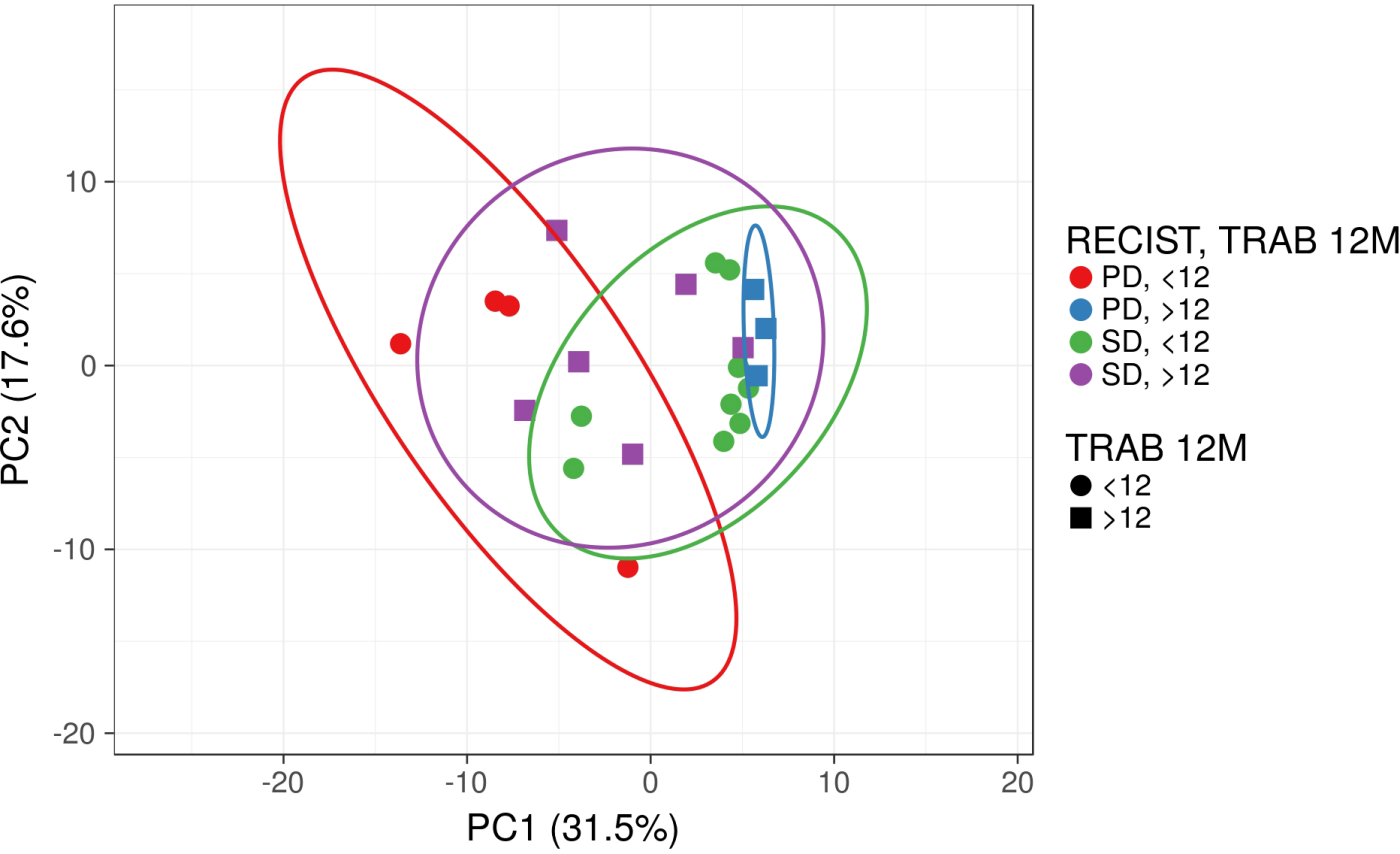
Principal Component Analysis (PCA) for Soft Tissue Sarcoma patients according to RECIST1.1 and duration of Trabectedin therapy. PD, Progression Disease; SD, Stable Disease; <12M, Trabectedin therapy for less than 12 months; ≥12M, Trabectedin therapy for 12 or more months.

Principal Component Analysis (PCA) was performed to correlate PFS with the duration of Trabectedin therapy using immunophenotyping, immune-related gene expression, and soluble proteome analysis data (**Figure 11**).

Although no clusters were separated from the other combinations, STS patients that progress disease (PD) with less than 12 months (<12M, in red) are distinct from those with 12 or more months (≥12M, in blue) of Trabectedin therapy.

STS patients with stable disease after Trabectedin therapy are similar in terms of the duration of Trabectedin therapy (green vs. purple).

**Figure 12:**
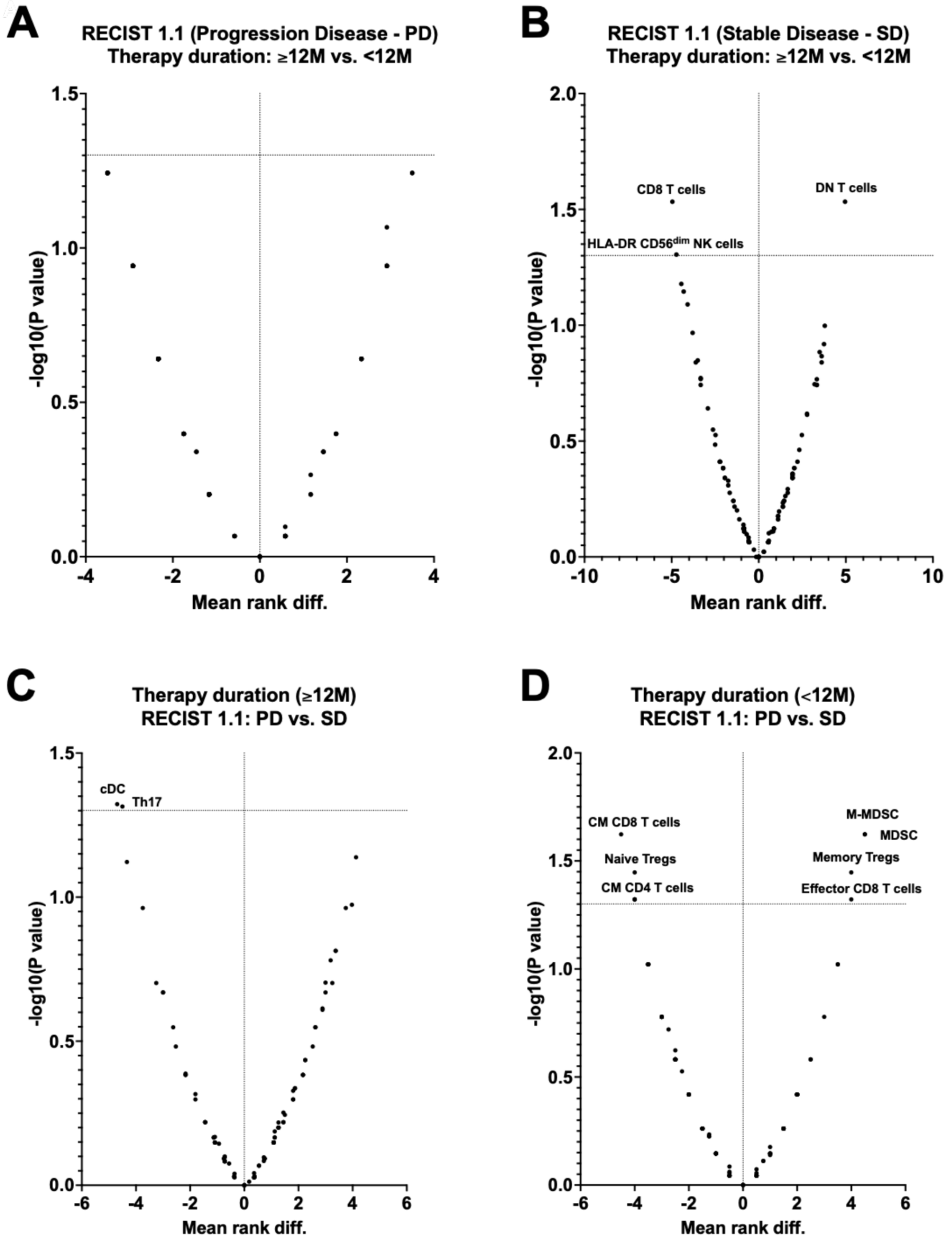
Volcano plots of immunophenotyping data of Soft Tissue Sarcoma patients according to RECIST1.1 and duration of Trabectedin therapy. **(A)** RECIST1.1 (Progression disease – PD) according to Therapy duration (≥12 vs. <12M); **(B)** RECIST1.1 (Stable disease - SD) according to Therapy duration (≥12 vs. <12M); **(C)** Therapy duration (<12M) according to RECIST1.1 (PD vs SD); **(D)** Therapy duration (≥12M) according to RECIST1.1 (PD vs SD). PD, Progression Disease; SD, Stable Disease; <12M, Trabectedin therapy for less than 12 months; ≥12M, Trabectedin therapy for 12 or more months.

A segregated analysis of RECIST1.1 and therapy duration for immunophenotyping data (**Figure 12A**) revealed significant differences for patients with SD according to therapy duration: increased DN T cells and decrease of CD8 T cells and activated HLA-DR CD56^dim^ NK cells (**Figure 12B**). Similarly, significant differences were observed for patients with <12M of therapy (**Figure 12C**) and ≥12M of trabectedin (**Figure 12D**) according to RECIST1.1.

**Figure 13:**
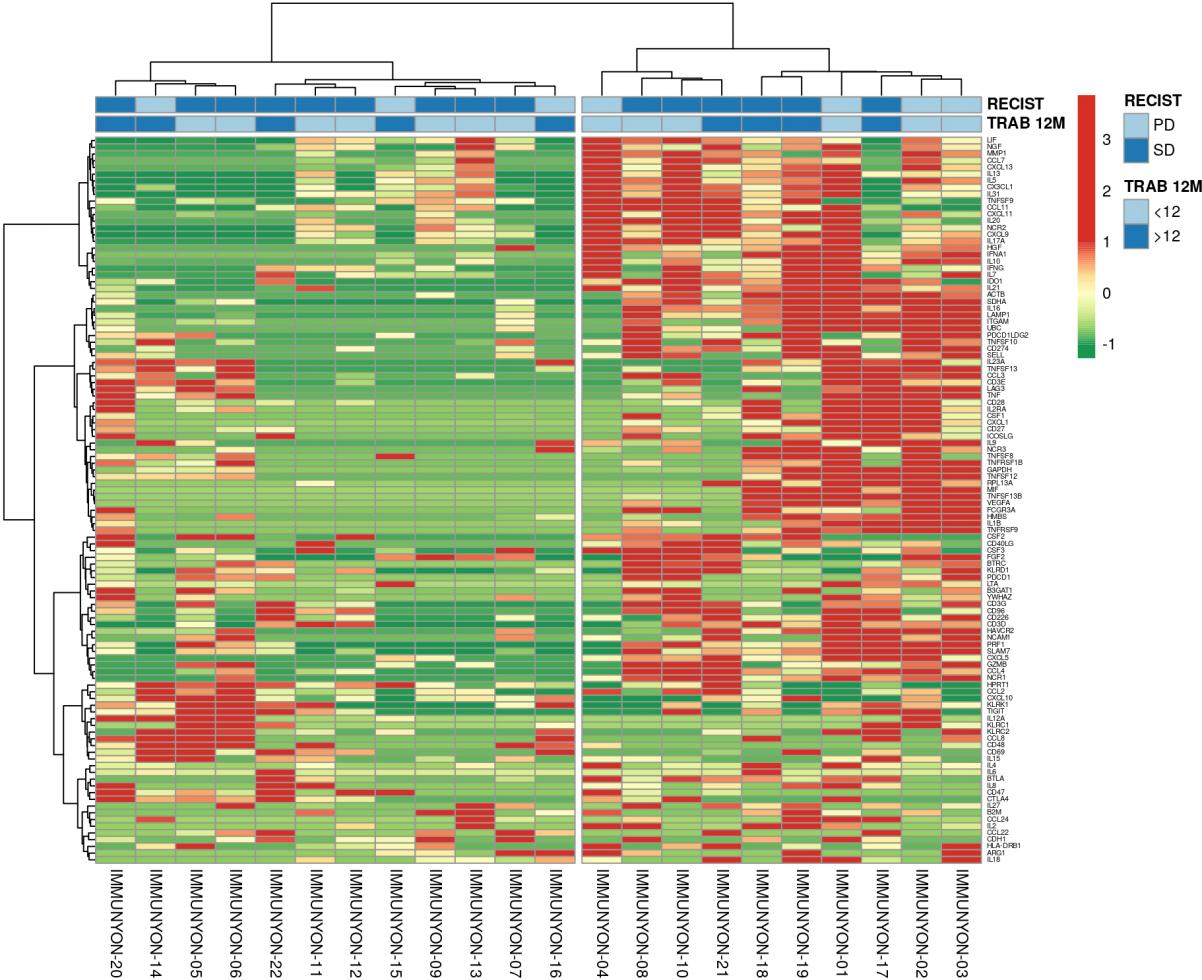
Heatmap of unsupervised clustering analysis for Soft Tissue Sarcoma patients according to RECIST1.1 and duration of Trabectedin therapy. PD, Progression Disease; SD, Stable Disease; <12M, Trabectedin therapy for less than 12 months; ≥12M, Trabectedin therapy for 12 or more months.

The ClustVis hierarchical clustering method (34) was applied to dimensions and observations resulted from the above immune-related parameters analyzed for STS patients in this study (**Figure 13**).

A heatmap clustering the multivariate data resulted in an unbiased identification of 2 clusters according to the immunophenotyping, immune-related gene expression, and soluble proteome analysis data with annotated RECIST1.1 and Trabectedin therapy duration.

### 3.4 Differential gene expression of BTRC, IFNA1, IL5, IL9 and LIF and survival in TCGA sarcoma cohort

**Figure 14:**
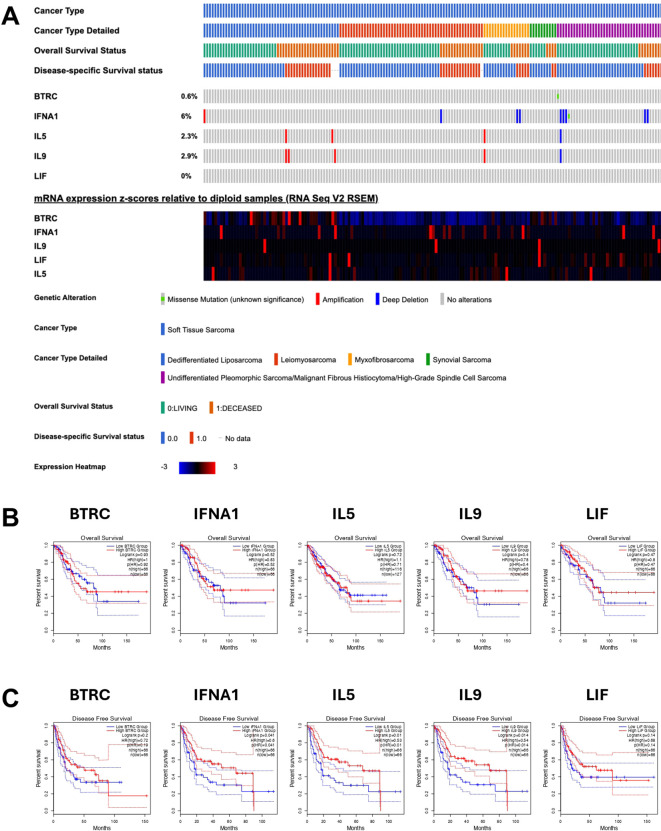
Oncoprint of clinical and genomic features of soft tissue sarcoma patients derived from TCGA sarcoma. **(A)** Oncoprint including mRNA expression z-scores relative to diploid samples **(B)** Overall survival and **(C)** PFS for BTRC, IFNA1, IL5, IL9 and LIF mRNAs from TCGA sarcoma dataset.

We evaluated the prognostic biomarkers suggested in this study for predictive power by accessing public databases. The sarcoma dataset originated from the genomic and expression analysis of 206 sarcoma samples within the TCGA Adult Soft Tissue Sarcomas cohort. A total of 174 samples with all relevant data available were selected for analysis. The gene characterized by the highest number of genomic or transcriptomic alterations (**Figure 14A**) was IFNA1 (altered in 8% of patients), followed by IL9 (altered in 2.9% of patients), IL5 (altered in 2.3% of patients) and BTRC (altered in 0.6% of patients). The most frequent IFNA1 alteration was deep deletion. Due to the limited number of sarcoma samples treated with trabectedin, which precluded a meaningful analysis, we shifted focus to examining differences in the expression levels of the five identified genes within the TCGA sarcoma cohort to explore potential correlations with survival outcomes. The expression levels of BTRC, IFNA1, IL5, IL9 and LIF genes were not significantly related to OS (**Figure 14B**). We found a significant relation between IFNA1 (Log-rank; p=0.02), IL5 (Log-rank; p=0.01), and IL9 (Log-rank; p=0.014) expression levels and disease-free survival (**Figure 14C**).

## 4 Discussion

This was a non-interventional study. The primary objective was to assess PFS in STS patients treated with trabectedin as measured by RECIST1.1 criteria. Immune monitoring of Trabectedin therapy in refractory STS patients aimed at the establishment of additional efficacy parameters of different therapeutic strategies.

More than one-third of STS patients achieved SD after Trabectedin therapy, in accordance with other studies (37). In this study, almost 40% of the patients receiving Trabectedin were analyzed for 12 or more months. Duration of Trabectedin therapy does not demonstrate a significant impact in PFS.

Regarding the immune monitoring associated with PFS, a significant decrease in eosinophils , increase of type 2 helper T cells (Th2) and decrease conventional dendritic cells (cDC) was associated with progression disease (PD) when compared to stable disease (SD). As far as the authors are aware, there is no description of those associations in the literature analyzing Trabectedin therapy in STS.

In this study, decreased beta-transducin repeat containing (BTRC) and increased interferon alpha-1 (IFNA1) and interleukin 9 (IL9) gene expression were significantly associated with a worse response to Trabectedin therapy. Several authors associated the pathways involving BTRC with osteosarcoma (38–40), but no studies described its involvement in STS. One study (41) referred to an enrichment of anti-tumor immune regulatory mechanisms, including interferon-gamma (IFN-γ) and interferon-alpha (IFN-α) responses in Ewing sarcoma. The studies analyzing IL-9 are scarce and result from side observations in cancer cell lines (42–44).

No soluble factors were found associated with PFS in STS patients treated with trabectedin in this study. Other investigators (45) described the chemokine CCL-2 and the cytokine IL-6 remarkedly reduced by Yondelis^®^ in monocytes, macrophages, TAM, and freshly isolated ovarian cells. The chemokine CCL2 is the major determinant of monocyte recruitment at tumor sites, whereas IL-6 is a growth factor for ovarian tumors.

The duration of Trabectedin therapy was also evaluated in this study. Although no significant alterations were observed in the complete blood count (CBC), several lymphocytes’ populations were significantly decreased in the first year of treatment, including exhausted B cells and activated T and NK cells. Again, this is the first study to describe this association in peripheral blood to trabectedin therapy.

Immune-related gene expression profiling revealed that LIF and IL5 mRNAs were significantly increased for the first 12 months of Trabectedin therapy. LIF was described as a super-enhancer-controlled regulator of stem cell-like properties in osteosarcoma (46). Another study on benign enchondromas and malignant chondrosarcomas (47) described also the presence of LIF, in contrast with its absence in control tissue.

Again, no soluble factors were found associated with duration of trabectedin therapy in this study.

Finally, Principal Component Analysis identified separated clusters of STS patients with PD according to the duration of Trabectedin therapy. An heatmap also revealed two clusters/signatures without discrimination of PFS (PD vs. SD) or duration of Trabectedin therapy (<12M vs. ≥12M).

In conclusion, the present study measured the impact of Trabectedin therapy in PFS by RECIST1.1. Several immune parameters (immune cells, immune-related genes, and soluble proteome) were associated with a better response to Trabectedin therapy. This study also allowed us to identify the short-term (<12M) and long-term (≥12M) effects of Trabectedin therapy. Early effects of Trabectedin (<12M) include the exhaustion of B cells, activation of T and NK cells, and increase of LIF gene expression associated with control of stemness properties of sarcoma cells.

The findings of this study must be interpreted within the context of several limitations. One of the primary limitations is the relatively small sample size, which may reduce the statistical power of the results and limit the generalizability of the findings. Additionally, the heterogeneity of the histotypes included in the study further complicates the interpretation of the results, as the immune response to trabectedin could vary across different sarcoma subtypes. The inclusion of diverse histological types may have introduced variability in treatment response and immune profile, potentially masking specific biomarkers or immune signatures that could be relevant for particular STS subgroups. While these limitations must be considered, the study provides valuable preliminary insights into the immune-related parameters associated with trabectedin therapy in STS. These findings lay the groundwork for future studies with larger, more homogeneous patient populations to validate the identified biomarkers and further elucidate their role in predicting treatment efficacy.

## Data Availability

The raw data supporting the conclusions of this article will be made available by the authors, without undue reservation.
