# Supplementary material for: Immune monitoring of trabectedin therapy in refractory soft tissue sarcoma patients - the IMMUNYON study
Source: Front Immunol. 2025 Feb 11;16:1516793. doi: 10.3389/fimmu.2025.1516793 (PMC11850243; doi:10.3389/fimmu.2025.1516793)
Supplement: Supplementary file 1 [file DataSheet1.pdf]

Supplementary Material

Supplementary file S1. Panels of fluorochrome-conjugated antibodies used in flow cytometry analysis

Fluorochrome	T cells	T_{reg} cells	Th1/Th2/Th17	B cells	DCs, Mo & NK	MDSC
**FITC**	PD-1	PD-1	PD-1	PD-1	PD-1	CD45
**PE**	CCR7	CD25	CXCR3	CD10	CD56	CD33
**PerCP-Cy5.5**	CD4	CD4	CD4	CD19	CD123	CD3/CD19/CD56
**PE-Cy7**	CD45RA	CCR4	CCR6	CD27	CD11c	CD15
**APC**	CD38	CD127	CD38	CD38	CD16	CD11b
**APC-H7**	CD8	CD45RO	CD8	CD20	CD3/CD19/CD20	CD16
**V450**	CD3	CD3	CD3	CD3	CD14	CD14
**V500**	HLA-DR	HLA-DR	HLA-DR	IgD	HLA-DR	HLA-DR

Legend: *FITC - Fluorescein isothiocyanate, PE - R-phycoerythrin, PerCP-Cy5.5 - peridinin chlorophyll protein-cyanine 5.5, PE-Cy7 - R-phycoerythrin – cyanine 7, APC – Allophycocyanin, AF647 – Alexa Fluor 647, APC-H7 – Allophycocyanin cyanine H7, V450 - Violet 450, PB – Pacific Blue 3-carboxy-6,8-difluoro-7-hydroxycoumarin, V500 – Violet 500.*

Supplementary file S2. Fluorochrome-conjugated monoclonal antibodies used in flow cytometry analysis.

Antibody	Conjugate	Clone	Brand	Cat#
CD3	V450	ICHT1	BD Horizon™	561416
CD3	APC-H7	SK7	BD Pharmingen™	560176
CD3	PerCp-Cy5.5	HIT3a	BioLegend®	300328
CD4	PerCp-Cy5.5	OKT4	BioLegend®	317428
CD8	APC-H7	HIT8a	BD™	641400
CD11b	APC	ICRF44	BioLegend®	301310
CD11c	PE/Cy7	B-ly6	BD Pharmingen™	561356
CD14	V450	MOP9	BD Horizon™	560349
CD15	PE/Cy7	HI98	BD Pharmingen™	560827
CD16	APC-Cy7	3G8	BioLegend®	302018
CD19	APC-H7	SJ2501	BD Pharmingen™	560177
CD19	PerCp-Cy5.5	HIB19	BioLegend®	302230
CD20	APC-H7	2H7	BD Pharmingen™	560734
CD24	FITC	ML5	BD Pharmingen™	555427
CD25	PE	M-A251	BD Pharmingen™	555432
CD27	PE/Cy7	M-T271	BD Pharmingen™	560609
CD33	PE	WM53	BD Pharmingen™	555450
CD38	APC	HIT2	BD Pharmingen™	555462
CD45	FITC	HI30	BD Pharmingen™	555482
CD45RA	PE/Cy7	5H9	BD Pharmingen™	561216
CD45RO	APC-H7	UCHL1	BD Pharmingen™	561137
CD56	PerCp-Cy5.5	HCD56	BD Pharmingen™	560842
CD56	PE	B159	BioLegend®	318306
CD123	PerCp-Cy5.5	6H6	BioLegend®	306016
CD127	AF647	HIL-7R-M21	BD Pharmingen™	558598
CD183 (CXCR3)	PE	IC6/CXCR3	BD Pharmingen™	550633
CD194 (CCR4)	PE/Cy7	IG1	BD™	577864
CD196 (CCR6)	PE/Cy7	11A9	BD Pharmingen™	560620
CD197 (CCR7)	PE	150503	BD Pharmingen™	560765
CD274 (PD-L1)	FITC	MIH1	BD Pharmingen™	558065
CD279 (PD-1)	FITC	MIH4	BD Pharmingen™	557860
CD279 (PD-1)	APC	MIH4	BD Pharmingen™	558694
CD279 (PD-1)	PE	MIH4	BD Pharmingen™	557946
HLA-DR	V500	G46-6	BD Horizon™	561224
IgD	V500	IAS6-2	BD Horizon™	561490

***Legend**: FITC - Fluorescein isothiocyanate, PE - R-phycoerythrin, PerCP-Cy5.5 - peridinin chlorophyll protein-cyanine 5.5, PE-Cy7 - R-phycoerythrin – cyanine 7, APC – Allophycocyanin, AF647 – Alexa Fluor 647, APC-H7 – Allophycocyanin cyanine H7, V450 - Violet 450, PB – Pacific Blue 3-carboxy-6,8-difluoro-7-hydroxycoumarin, V500 – Violet 500.*

Supplementary file S3 – Representative gating strategy used to analyze the data obtained using multiparametric flow cytometry, which identified the immune populations present in peripheral blood samples. A) Gating strategy for T cells, B cells, NK cells, dendritic cells, and monocytes. Lymphocytes were discriminated based on SSC-A and FSC-A and doublets excluded [1a – 1c]. Next, T cells were selected by positivity to CD3 [2]. T cells were plotted on a CD4 versus CD8 plot to identify CD4 T and CD8 T cells, as well as double positive ($CD4^+CD8^+$) and negative ($CD4^-CD8^-$) cells [2a]. Gated on CD4 T [2b1] and CD8 T cells [2b2], naïve cells ($CD45RA^+$ $CCR7^+$), central memory cells ($CD45^-$ $CCR7^+$), effector memory cells ($CD45RA^-$ $CCR7^-$) and effector cells ($CD45RA^+$ $CCR7^-$) were discriminated. Within CD4 T cells, expression of CX3CR1 or CCR6 mAbs were used to differentiate Th1 cells ($CX3CR1^+$ $CCR6^-$), Th17 ($CX3CR1^-$ $CCR6^+$) and Th2 cells ($CX3CR1^-$ $CCR6^-$) [3a]. Activation status of lymphocytes subpopulations were accessed by the presence of the activation marker HLA-DR, representative histogram for HLA-DR expression in Th1 cells [3b]. Treg cells, gated on CD4 T cells were identified by lower or absent expression of CD127 and expression of CD25 and CCR4 [4a – 4c]. The anti-CD45RO mAb were used to discriminate between naïve ($CD45RO^-$) and memory ($CD45RO^+$) Treg cells [4d]. Inhibition status of lymphocytes subpopulations were accessed by the presence of the inhibitory marker PD-1, representative histogram for PD-1 expression in Treg cells [4e]. Gated on lymphocytes, B cells were selected by positivity to CD19 [5a]. B cells were plotted in a CD27 versus IgD plot to identify naïve cells (IgD^+ $CD27^-$), pre-switch memory cells (IgD^+ $CD27^+$), switch memory cells (IgD^- $CD27^+$) and exhausted cells (IgD^- $CD27^-$) [5b]. Gated on naïve B cells, plasmablasts were identified by positivity to CD38 and negativity to CD24 [5c]. Within lymphocytes, NK cells were selected by positivity to CD56 and absence of CD3 and NKT-like cells were identified by the positivity for both [6a – 6d]. Peripheral blood mononuclear cells were selected in a SSC-A and FSC-A plot, then cells positive for CD3, CD19, CD20, CD56 and CD14 mAbs were excluded and the anti-HLA-DR mAb was used as a specific marker to identify the DC [7a – 7c]. CD11c and CD123 identified $CD11c^+$ $CD123^{+/-}$ classical DC (cDC, $CD11c^+$ $CD123^{+/-}$) and plasmacytoid DC (pDC, $CD11c^-$ $CD123^+$) [7d]. B) Gating strategy myeloid-derived suppressor cells (MDSC). First, we selected the positive cells for the CD45 marker and then we proceeded with the exclusion of cells positive for: CD16, HLA-DR, CD3, CD56, and CD19 [1 – 4]. MDSC were then identified by the expression of CD33 and CD11b [5]. Gated on MDSC, three subpopulations were discriminated based on the combination of the anti-CD14 and anti-CD15 mAbs, early-MDSC (e-MDSC: $CD14^-$, $CD15^-$), monocytic-MDSC (M-MDSC: $CD14^+$, $CD15^{-/+}$) and polymorphonuclear-MDSC (PMN-MDSC: $CD14^-$, $CD15^+$) [6]. All samples were analyzed with FlowJo v.10.7 software (BD Biosciences, OH, USA).

A

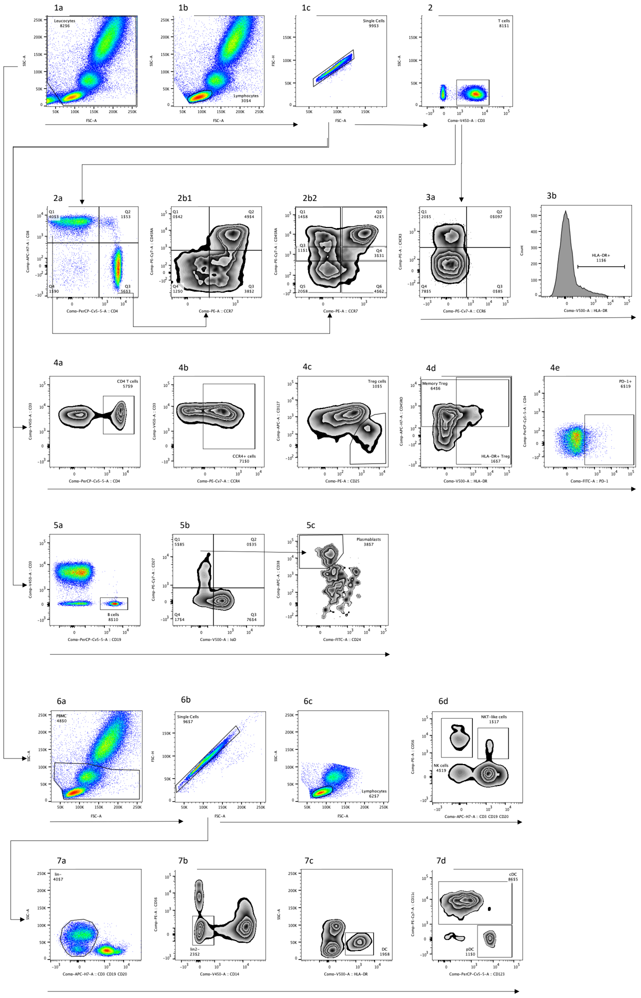

B

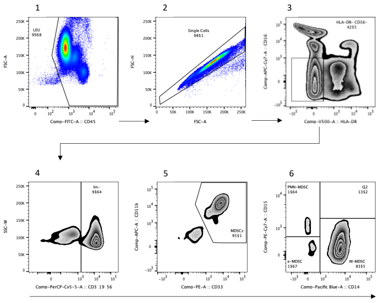

Supplementary file S4 – Oligonucleotide primers used to preform gene expression analysis by real-time PCR.

Gene name	NCBI Gene ID	GenBank ID	Primer Forward	Primer Reverse	Ref or PrimerBank ID
Reference genes					
ACTB	60	NM_001101	CTGGAACGGTGAAGGTGACA	AAGGGACTTCCTGTAACAATGCA	(Vandesompele 2002)
B2M	567	NM_004048	TGCTGTCTCCATGTTTGATGTATCT	TCTCTGCTCCCCACCTCTAAGT	(Vandesompele 2002)
GAPDH	2597	NM_002046	TGCACCACCAACTGCTTAGC	GGCATGGACTGTGGTCATGAG	(Vandesompele 2002)
HMBS	3145	NM_000190	GGCAATGCGGCTGCAA	GGGTACCCACGCGAATCAC	(Vandesompele 2002)
HPRT1	3251	NM_000194	TGACACTGGCAAAACAATGCA	GGTCCTTTTCACCAGCAAGCT	(Vandesompele 2002)
RPL13A	23521	NM_012423	CCTGGAGGAGAAGAGGAAAGAGA	TTGAGGACCTCTGTGTATTTGTCAA	(Vandesompele 2002)
SDHA	6389	NM_004168	TGGGAACAAGAGGGCATCTG	CCACCACTGCATCAAATTCATG	(Vandesompele 2002)
UBC	7316	M26880	ATTTGGGTCGCGGTTCTTG	TGCCTTGACATTCTCGATGGT	(Vandesompele 2002)
YWHAZ	7534	NM_003406	ACTTTTGGTACATTGTGGCTTCAA	CCGCCAGGACAAACCAGTAT	(Vandesompele 2002)
Genes of interest					
ARG1	383	NM_000045	GTGGAAACTTGCATGGACAAC	AATCCTGGCACATCGGGAATC	346986433c1
B3GAT1	27087	NM_018644	CCTGGCGTGGTCTACTTCG	GCAGGTTGACGGCAAATCC	77695913c1
BTLA	151888	NM_001085357	CATCTTAGCAGGAGATCCCTTTG	GACCCATTGTCATTAGGAAGCA	145580618c1
BTRC	8945	NM_003939	CCAGACTCTGCTTAAACCAAGAA	GGGCACAATCATACTGGAAGTG	379030597c1
CCL11	6356	NM_002986	CCCCTTCAGCGACTAGAGAG	TCTTGGGGTCGGCACAGAT	22538399c1
CCL2	6347	NM_002982	CAGCCAGATGCAATCAATGCC	TGGAATCCTGAACCCACTTCT	4506841a1
CCL22	6367	NM_002990	ATCGCCTACAGACTGCACTC	GACGGTAACGGACGTAATCAC	300360575c1
CCL24	6369	NM_002991	ACATCATCCCTACGGGCTCT	CTTGGGGTCGCCACAGAAC	22165426c1
CCL3	6348	NM_002983	AGTTCTCTGCATCACTTGCTG	CGGCTTCGCTTGGTTAGGAA	4506843a1
CCL4	6351	NM_002984	CTGTGCTGATCCCAGTGAATC	TCAGTTCAGTTCCAGGTCATACA	4506845a1
CCL7	6354	NM_006273	GACAAGAAAACCCAAACTCCAAAG	TCAAAACCCACCAAAATCCA	(Wang 2021)
CCL8	6355	NM_005623	TGGAGAGCTACACAAGAATCACC	TGGTCCAGATGCTTCATGGAA	22538815c1
CD28	940	NM_001243078	CTATTTCCCGGACCTTCTAAGCC	GCGGGGAGTCATGTTCATGTA	340545509c1
CD27	939	NM_001242	CAGAGAGGCACTACTGGGCT	CGGTATGCAAGGATCACACTG	117422442c1
CD274	29126	NM_014143	TGGCATTTGCTGAACGCATTT	TGCAGCCAGGTCTAATTGTTTT	292658763c1
CD276	80381	NM_001024736	CTTGTTCGATGTTCACAGCG	GCCGTAGAGCTGTCTTGGATC	(Fabien 2017)
CD3D	915	NM_001040651	ACTGGCTACCCTTCTCTCG	CCGTTCCCTCTACCCATGTGA	98985800c1
CD3E	916	NM_000733	CCTCTTATCAGTTGGCGTTTGG	TTCAGTGACAGGTGATCCTCA	166362733c1
CD3G	917	NM_000073	TGGCCCAGTCAATCAAAGGAA	CAAGTCAGAAGTACCGAACCATC	166362738c1
CD40LG	959	NM_000074	ACATACAACCAAACTTCTCCCCG	GCAAAAAGTGCTGACCCAATCA	58331233c1
CD47	961	NM_001777	AGAAGGTGAAACGATCATCGAGC	CTCATCCATACCACCGGATCT	68223312c1
CD48	962	NM_001778	AGGTTGGGATTCGTGTCTGG	AGTTGTTTGTAGTTCTCAGGCAG	365733591c1
CD69	969	NM_001781	ATTGTCCAGGCCAATACACATT	CCTCTCTACCTGCGTATCGTTTT	221554485c1
CD96	10225	NM_198196	CAAACACAGACAGTAGGCTTCTT	GGGGATGATAGACAGCAATCAG	93141044c1
CDH1	999	NM_004360	CGAGAGCTACACGTTCACGG	GGGTGTCGAGGGAAAAATAGG	169790842c1
CSF1	1435	NM_172210	TGGCGAGCAGGAGTATCAC	AGGTCTCCATCTGACTGTCAAT	166235149c1
CSF2	1437	NM_000758	TCCTGAACCTGAGTAGAGACAC	TGCTGCTTGTAGTGGCTGG	371502128c1
CSF3	1440	NM_001178147	GCTGCTTGAGCCAACTCCATA	GAACGCGGTACGACACCTC	296011056c1
CTLA4	1493	NM_005214	GCCCTGCACTCTCCTGTTTTT	GGTTGCCGCACAGACTTCA	339276048c1
CX3CL1	6376	NM_002996	ACCACGGTGTGACGAAATG	TGTTGATAGTGGATGAGCAAAGC	54111253c1
CXCL1	2919	NM_001511	GCGCCCAAACCGAAGTCATA	ATGGGGGATGCAGGATTGAG	(Zhou 2021)
CXCL10	3627	NM_001565	GTGGCATTCAAGGAGTACCTC	TGATGGCCTTCGATTCTGGATT	323422857c1

CXCL11	6373	NM_005409	GACGCTGTCTTTGCATAGGC	GGATTTAGGCATCGTTGTCCTTT	307611978c1
CXCL13	10563	NM_006419	GCTTGAGGTGTAGATGTGTCC	CCCACGGGGCAAGATTTGAA	194733765c1
CXCL5	6374	NM_002994	AGCTGCGTTGCGTTTGTTTAC	TGGCGAACACTTGCAGATTAC	41872613c1
CXCL9	4283	NM_002416	CCAGTAGTGAGAAAGGGTCGC	AGGGCTTGGGGCAAATTGTT	4505186c1
FCGR3A	2214	NM_000569	CCTCCTGTCTAGTCGGTTTGG	TCGAGCACCCTGTACCATTGA	24429586a1
FGF2	2247	NM_002006	AGAAGAGCGACCCTCACATCA	CGGTTAGCACACACTCCTTTG	153285460c1
GZMB	3002	NM_004131	CCCTGGGAAAACACTCACACA	GCACAACTCAATGGTACTGTCG	221625527c1
HAVCR2	84868	NM_032782	CTGCTGCTACTACTTACAAGGTC	GCAGGGCAGATAGGCATTCT	354681988c1
HGF	3082	NM_001010931	GCTATCGGGGTAAAGACCTACA	CGTAGCGTACCTCTGGATTGC	58533162c1
HLA-DRB1	3123	AJ297586	GAGCAGGTTAAACATGAGTGTCA	CTCTCCACAACCCCGTAGT	15387629a1
ICOSLG	23308	NM_015259	GCAGCCTTCGAGCTGATACTC	GTTTTCGACTCACTGGTTTGC	58331247c1
IDO1	3620	NM_002164	GCCAGCTTCGAGAAAGAGTTG	ATCCCAGAACTAGACGTGCAA	323668304c1
IFNA1	3439	NM_024013	GCCTCGCCCTTTGCTTTACT	CTGTGGGTCTCAGGGAGATCA	13128950a1
IFNG	3458	NM_000619	TCGGTAACTGACTTGAATGTCCA	TCGCTTCCCTGTTTTAGCTGC	56786137c1
IL10	3586	NM_000572	GACTTTAAGGGTTACCTGGGTTG	TCACATGCGCCTTGATGTCTG	24430216c1
IL12A	3592	NM_000882	CCTTGCACTTCTGAAGAGATTGA	ACAGGGCCATCATAAAAGAGGT	325974478c1
IL13	3596	NM_002188	CCTCATGGCGCTTTTGTTGAC	TCTGGTTCTGGGTGATGTTGA	26787977c1
IL15	3600	NM_172175	TTGGGAACCATAGATTTGTGCAG	GGGTGAACATCACTTTCCGTAT	26787986a1
IL16	3603	NM_172217	GCCGAAGACCCTTGGGTTAG	GCTGGCATTGGGCTGTAGA	289063450c1
IL17A	3605	NM_002190	TCCCACGAAATCCAGGATGC	GGATGTTCAGGTTGACCATCAC	27477085c1
IL18	3606	NM_001562	TCTTCATTGACCAAGGAAATCGG	TCCGGGGTGCATTATCTCTAC	342349317c1
IL1B	3553	NM_000576	ATGATGGCTTATTACAGTGGCAA	GTCGGAGATTCGTAGCTGGA	27894305c1
IL2	3558	M22005	TACAAGAACCCGAAACTGACTCG	ACATGAAGGTAGTCTCACTGCC	386818a1
IL20	50604	NM_018724	ATGAAAGCCTCTAGTCTTGCCT	GCCCCGTATCTCAGAAAATCC	50845426c1
IL21	59067	NM_021803	TAGAGACAAACTGTGAGTGGTCA	GGGCATGTTAGTCTGTGTTTCTG	365733583c1
IL23A	51561	NM_016584	CTCAGGGACAACAGTCAGTTC	ACAGGGCTATCAGGGAGCA	28144902c1
IL2RA	3559	NM_000417	GTGGGGACTGCTCACGTTC	CCCGCTTTTTATTCTGCGGAA	269973860c1
IL3	3562	NM_000588	CAGACAACGCCCTTGAAGACA	GCCCTGTTGAATGCCTCCA	28416914c1
IL31	386653	NM_001014336	CACGTTGCCCGTCCGTTTA	TCTTCGAGAGGGACTGTAATTCC	62122910c1
IL4	3565	NM_000589	CCAACTGCTTCCCCCTCTG	TCTGTTACGGTCAACTCGGTG	4504669a1
IL5	3567	NM_000879	TGGAGCTGCCTACGTGTATG	TTCGATGAGTAGAAAGCAGTGC	28559032c1
IL6	3569	NM_000600	ACTCACCTCTTCAGAACGAATTG	CCATCTTTGGAAGGTTCAGGTTG	224831235c1
IL7	3574	NM_000880	TTGGACTTCCTCCCCTGATCC	TCGATGCTGACCATTAGAACAC	4504677a1
IL8	3576	NM_000584	TTTTGCCAAGGAGTGCTAAAGA	AACCCTCTGCACCCAGTTTTC	10834978a1
IL9	3578	NM_000590	CTCTGTTTGGGCATTCCCTCT	GGGTATCTTGTTTGCATGGTGG	10834980a1
ITGAM	3684	NM_001145808	GCCTTGACCTTATGTCATGGG	CCTGTGCTGTAGTCGCACT	224831238c1
KLRC1	3821	NM_007328	AGCTCCATTTAGCAACTGAACA	CAACTATCGTTACCACAGAGGC	283046824c1
KLRC2	3822	NM_002260	GCCAGCATTTTACCTTCCTCA	ACTGCACAGTTAAGTTCAGCAT	4504883a1
KLRD1	3824	NM_007334	CAGGACCCAACATAGAACTCCA	GGAAATGAAGTAACAGTTGCACC	167614494c1
KLRF1	51348	NM_016523	TACTGGGAATATCTGGAACCGT	TTGAGCCATTCTGATTGGCAT	7705573c1
LAG3	3902	NM_002286	GCGGGGACTTCTCGCTATG	GGCTCTGAGAGATCCTGGGG	167614499c1
LAMP1	3916	NM_005561	TCTCAGTGAACTACGACACCA	AGTGTATGTCCTCTTCCAAAAGC	112380627c1
LIF	3976	NM_002309	CCAACGTGACGGACTTCCC	TACACGACTATGCGGTACAGC	380418322c1
LTA	4049	NM_000595	ATGACACCACCTGAACGTCTC	CTCTCCAGAGCAGTGAGTTCT	6806892c1
MIF	4282	NM_002415	GAACAACTCCACCTTCGCCT	CCGTTTATTTCTCCCCACCA	(Yao 2016)

MMP1	4312	NM_002421	AAAATTACACGCCAGATTTGCC	GGTGTGACATTACTCCAGAGTTG	225543092c1
NCAM1	4684	NM_001076682	GGCATTTACAAGTGTGTGGTTAC	TTGGCGCATTCTTGAACATGA	336285437c1
NCR1	9437	NM_001242357	TGGACCCGAAGTGATCTCG	TCCTTGAGCAGTAAGAACATGC	334358898c1
NCR2	9436	NM_004828	GGCTCTCAGGCACAATCCAAG	GCTGAAGCCTCCTTACACCA	153945781c1
NCR3	259197	NM_001145467	CCCCTGAGATTCGTACCCTG	CTCCACTCTGCACACGTAGAT	224586864c1
PDCD1	5133	NM_005018	CCAGGATGGTTCTTAGACTCCC	TTTAGCACGAAGCTCTCCGAT	167857791c1
PDCD1LG2	80380	NM_025239	ATTGCAGCTTCACCAGATAGC	AAAGTTGCATTCCAGGGTCAC	190014604c1
PRF1	5551	NM_005041	GGCTGGACGTGACTCCTAAG	CTGGGTGGAGGCGTTGAAG	133908619c1
SELL	6402	NM_000655	ACCCAGAGGGACTTATGGAAC	GCAGAATCTTCTAGCCCTTTGC	262206314c1
SLAMF7	57823	NM_021181	ACCCTCATCTATATCCTTTGGCA	CACCAACGGAACCGACCAG	19923571c1
TIGIT	201633	NM_173799	TCTGCATCTATCACACCTACCC	CCACCACGATGACTGCTGT	256600227c1
TNF	7124	NM_000594	CCTCTCTCTAATCAGCCCTCTG	GAGGACCTGGGAGTAGATGAG	25952110c1
TNFRSF1B	7133	NM_001066	CGGGCCAACATGCAAAAGTC	CAGATGCGGTTCTGTTCCC	23312365c1
TNFRSF9	3604	NM_001561	AGCTGTTACAACATAGTAGCCAC	GGACAGGGACTGCAAATCTGAT	315259099c1
TNFSF10	8743	NM_001190942	TGCGTGCTGATCGTGATCTTC	GCTCGTTGGTAAAGTACACGTA	300193031c1
TNFSF12	8742	NM_003809	GAGGGGAAGGCTGTCTACCT	GAACCTGGAAGAGTCCGAAGTA	23510442c1
TNFSF13	8741	NM_172088	CTCTGCTGACCCAACAAACAG	GGAGGTGGCGTTAATGGGAAC	23510442c1
TNFSF13B	10673	NM_001145645	GGGAGCAGTCACGCCTTAC	GATCGGACAGAGGGGCTTT	26051248a1
TNFRSF8	943	NM_001243	TCCACGGAGCACACCAATAAC	ACTGAGAGCATGACATCGCTG	325053721c1
TNFSF9	8744	NM_003811	GGCTGGAGTCTACTATGTCTTCT	ACCTCGGTGAAGGGAGTCC	68348710c1
TSLP	85480	NM_138551	ATGTTCGCCATGAAAACTAAGGC	GCGACGCCACAATCCTTGTA	209954675c1
VEGFA	7422	NM_001171627	AGGGCAGAATCATCACGAAGT	AGGGTCTCGATTGGATGGCA	372466598c1

Supplementary file S5. Analytes analyzed in ProcartaPlex Human Immune Monitoring 65-Plex Panel

Analyte	Bead Number	[Std1] (pg/mL)	LLOQ (pg/ml)	ULOQ (pg/ml)
APRIL	88	452300	110	452300
BAFF	86	13000	3.17	13000
BLC	29	46400	11	46400
bNGF	55	22200	5.42	22200
CD30	84	38600	9.42	38600
CD40-Ligand	74	42400	10	42400
ENA-78 (LIX)	82	37500	9.16	37500
Eotaxin	33	6150	1.5	6150
Eotaxin-2	30	15000	15	15000
Eotaxin-3	49	6850	1.67	6850
FGF-2	75	45000	11	45000
Fractalkine	59	9250	2.26	9250
G-CSF/CSF-3	42	52500	13	52500
GM-CSF	44	59700	15	59700
Gro-alpha/KC	61	16700	4.08	16700
HGF	46	21600	5.27	21600
IFN-alpha	48	29500	7.2	29500
IFN-gamma	43	54000	13	54000
IL-10	28	7350	1.79	7350
IL-12p70	34	26900	6.57	26900
IL-13	35	20000	4.88	20000
IL-15	65	12000	2.93	12000
IL-16	70	63300	15	63300
IL-17A	36	15800	3.86	15800
IL-18	66	39500	9.64	39500
IL-1alpha	62	7100	1.73	7100
IL-1beta	18	25600	6.25	25600
IL-2	19	30700	7.5	30700
IL-20	81	58900	14	14725
IL-21	72	38700	9.45	38700
IL-22	76	67700	17	67700
IL-23	63	54400	13	54400
IL-27	14	51700	13	51700

Analyte	Bead Number	[Std1] (pg/mL)	LLOQ (pg/ml)	ULOQ (pg/ml)
IL-2R	9	397600	97	397600
IL-3	73	112400	27	112400
IL-31	37	78900	19	78900
IL-4	20	44600	11	44600
IL-5	21	29900	7.3	29900
IL-6	25	58100	14	58100
IL-7	26	3100	0.76	3100
IL-8	27	11700	2.86	11700
IL-9	52	32600	7.96	32600
IP-10	22	8200	2	8200
I-TAC	57	42900	10	42900
LIF	15	16400	4	16400
MCP-1	51	10700	2.61	10700
MCP-2	8	4150	1.01	1038
MCP-3	68	16200	16	16200
M-CSF	67	57700	14	57700
MDC/CCL22	87	56500	14	56500
MIF	53	3800	0.93	3800
MIG	69	30200	7.37	30200
MIP-1alpha	12	14500	3.54	3625
MIP-1beta	47	34300	8.37	8575
MIP-3alpha	56	39600	9.67	39600
MMP-1	64	17200	4.2	17200
SCF	39	18900	4.61	18900
SDF-1alpha	13	185400	45	185400
TNF-alpha	45	51600	13	51600
TNF-beta	54	23600	5.76	23600
TNF-RII	85	10700	2.61	10700
TRAIL	58	13700	3.34	13700
TSLP	80	19500	4.76	19500
Tweak	97	393900	96	393900
VEGF-A	78	27400	6.69	27400

Supplementary file S6. Analytes analyzed in ProcartaPlex Human Immuno-Oncology Checkpoint Panel 1, 2 and 3.

ProcartaPlex Human Immuno-Oncology Checkpoint Panel 1 (14-Plex)

Analyte	Bead Number	[Std1] (pg/mL)	LLOQ (pg/ml)	ULOQ (pg/ml)
BTLA	52	492500	120	49250
GITR	57	85500	21	85500
HVEM	36	59700	15	59700
IDO	46	13200	3.22	13200
LAG-3	47	43700	11	43700
PD-1	65	30000	7.32	30000
PD-L1	66	14500	3.54	14500
PD-L2	67	189300	46	18930
TIM-3	14	303700	74	30370
CD28	15	132800	32	13280
CD80	61	150700	37	15070
CD137/4-1BB	26	47400	12	47400
CD27	27	23900	5.83	23900
CD152/CTLA4	33	34700	8.47	34700

ProcartaPlex Human Immuno-Oncology Checkpoint Panel 2 (14-Plex)

Analyte	Bead Number	[Std1] (pg/mL)	LLOQ (pg/ml)	ULOQ (pg/ml)
MICA	18	53100	13	53100
MICB	21	30000	7.32	30000
Nectin-2	29	152800	37	15280
NT5E (CD73)	30	184800	45	18480
PVR (CD155)	56	222500	54	22250
Siglec-7	12	63000	62	63000
Siglec-9	13	7600	1.86	7600
ULBP-1	73	584700	143	58470
ULBP-3	77	81000	79	81000
Tactile (CD96)	35	291000	71	29100
E-Cadherin	44	148600	36	14860
Arginase	51	50000	12	50000
ULBP-4	78	337100	82	33710
Perforin	53	258700	63	258700

ProcartaPlex Human Immuno-Oncology Checkpoint Panel 3 (10-Plex)

Analyte	Bead Number	[Std1] (pg/mL)	LLOQ (pg/ml)	ULOQ (pg/ml)
B7-H6	42	485400	119	48540
CD134 (OX40)	55	30800	7.52	30800
CD276 (B7-H3)	72	584700	571	58470
CD47 (IAP)	74	25100	6.13	25100
CD48 (BLAST-1)	19	116400	28	11640
Galectin-9	38	9100	2.22	9100
ICOS Ligand (B7-	34	37000	9.03	37000
S100A8/A9	76	249200	61	24920
TIMD-4	39	248600	61	24860
VISTA (B7-H5)	64	35400	8.64	35400
